# The Development of Poly(lactic acid) (PLA)-Based Blends and Modification Strategies: Methods of Improving Key Properties towards Technical Applications—Review

**DOI:** 10.3390/ma17184556

**Published:** 2024-09-17

**Authors:** Jacek Andrzejewski, Subhasis Das, Vitali Lipik, Amar K. Mohanty, Manjusri Misra, Xiangyu You, Lay Poh Tan, Boon Peng Chang

**Affiliations:** 1 Institute of Materials Technology, Poznan University of Technology, Piotrowo 3 Str., 61-138 Poznan, Poland; jacek.andrzejewski@put.poznan.pl; 2 School of Materials Science & Engineering, Nanyang Technological University, 50 Nanyang Avenue, Singapore 639798, Singapore; subhasis.das@ntu.edu.sg (S.D.); vitali@ntu.edu.sg (V.L.); 3 School of Engineering, University of Guelph, 50 Stone Road East, Guelph, ON N1G 2W1, Canada; mohanty@uoguelph.ca (A.K.M.); mmisra@uoguelph.ca (M.M.); 4 Bioproducts Discovery and Development Centre, Department of Plant Agriculture, Crop Science Building, University of Guelph, 50 Stone Road East, Guelph, ON N1G 2W1, Canada; 5 College of Bioresources Chemical and Materials Engineering, Shaanxi University of Science and Technology, Xi’an 710021, China; xyyou@sust.edu.cn

**Keywords:** poly(lactic acid), polymer blends, multiphase structures, durability, engineering application

## Abstract

The widespread use of poly(lactic acid) (PLA) from packaging to engineering applications seems to follow the current global trend. The development of high-performance PLA-based blends has led to the commercial introduction of various PLA-based resins with excellent thermomechanical properties. The reason for this is the progress in the field of major PLA limitations such as low thermal resistance and poor impact strength. The main purpose of using biobased polymers in polymer blends is to increase the share of renewable raw materials in the final product rather than its possible biodegradation. However, in the case of engineering applications, the focus is on achieving the required properties rather than maximizing the percentage of biopolymer. The presented review article discusses the current strategies to optimize the balance of the key features such as stiffness, toughness, and heat resistance of PLA-based blends. Improving of these properties requires molecular structural changes, which together with morphology, crystallinity, and the influence of the processing conditions are the main subjects of this article. The latest research in this field clearly indicates the high potential of using PLA-based materials in highly demanding applications. In the case of impact strength modification, it is possible to obtain values close to 800 J/m, which is a value comparable to polycarbonate. Significant improvement can also be confirmed for thermal resistance results, where heat deflection temperatures for selected types of PLA blends can reach even 130 °C after modification. The modification strategies discussed in this article confirm that a properly conducted process of selecting the blend components and the conditions of the processing technique allows for revealing the potential of PLA as an engineering plastic.

## 1. Introduction

### 1.1. Bioplastics, Present Status, and Challenges

The rapid growth of the bioplastics industry has been primarily propelled by the widespread production of film and packaging, driven by the potential for biodegradation/composting of such waste [1,2]. Traditionally, the mechanical properties, thermal resistance, and durability of these polymers held little significance as their primary utility lay in rapid decomposition. However, a paradigm shift is underway, with an increasing number of industries aligning their technological processes with the principles of the circular economy [3,4]. This necessitates a departure from prevailing resource management policies, particularly evident in plastics production, which heavily relies on crude oil [5,6,7].

Despite the rising interest in biobased materials, only a few select among them are entirely derived from renewable sources, with polylactic acid (PLA) emerging as the frontrunner [8]. However, it is worth noting that the “bio” prefix often pertains solely to biodegradability, with the synthesis of many purportedly “biobased” polymers relying partially or wholly on non-renewable resources [9,10,11,12]. While initially popularized in packaging, the appeal of biobased materials has extended to manufacturers beyond this sector. Nonetheless, challenges persist, particularly in meeting the rigorous performance standards demanded by industries such as electronics, machinery, and automotive [13,14]. This performance gap is not unique to biobased polymers, as even traditional polymers often necessitate blending to meet specific property criteria, driven not only by technical requirements but also economic considerations [15,16]. Unfortunately, many of these practices prioritize economic gains for manufacturers over environmental benefits.

**Figure 1:**
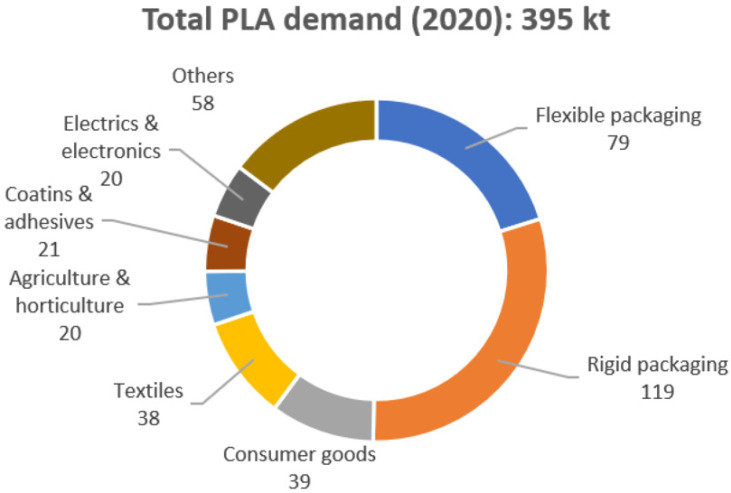
Estimated global PLA demand by end use (2020). Adapted from Ref. [35]. Copyright 2023 MDPI.

Figure 1 presents the estimated global demand for PLA by end use in 2020. PLA has gained prominence in the packaging industry due to its resemblance to poly(ethylene terephthalate) (PET), facilitating ease of processing, alongside its biodegradability and 100% biobased content, making it the most sought-after bioplastic globally. 

It is widely recognized that biopolymers are increasingly being promoted as sustainable packaging materials. However, this positive trend is often overshadowed by public perceptions that biopolymers generally exhibit poor mechanical properties. This perception is largely due to the common use of biodegradable films made from polybutylene adipate terephthalate (PBAT) or polycaprolactone (PCL) blended with thermoplastic starch (TPS), which are known for their limited mechanical strength [17,18,19,20].

The main goal of the paper, as previously discussed, was to present the main strategies for the modification of PLA-based materials, especially in the context of industrial modification methods through the preparation of polymer blends, composite systems, or hybrid methods, including thermal treatment. For most of the cited research examples, the modification involved the processing of materials commercially available on the market and not the preparation of new methods of polymerization of PLA or its copolymers [21,22]. For the same reasons, the presented review does not cover the broad field of PLA application in the medical industry, where this polymer finds numerous applications, both in traditional surgery and modern regenerative medicine therapies [23,24,25,26]. Currently, the greatest increase in interest in the use of PLA is due to 3D printing techniques, in particular the most popular of them, the material extrusion method (MEX) using filament; these techniques are also known as FDM (fused deposition modeling) and FFF (fused filament fabrication) [27,28,29,30]. It is worth noting, however, that in the case of medical applications, as well as 3D printing, the key physicochemical properties that determine their use are also their greatest obstacle from the perspective of technical applications [31,32,33,34]. In the case of the medical industry, it is the ability to degrade and resorb in intracorporeal conditions; for the area of 3D printing, low crystallinity limits the shrinkage of manufactured products. As a consequence, the traits that make them successful in relatively niche industries may be obstacles to their success in technical applications, such as the automotive, electrical, and machinery industries.

### 1.2. Requirements for Engineering Materials

The requirements for polymeric materials depend on many factors. For engineering plastics, it is essential that they meet the basic mechanical and physical requirements that determine the suitability of a given polymer in the chosen application field. However, the popularity and cost–effectiveness of using a specific polymer depends often on the sum of many purely technological factors, depending firstly on the processing technology of the final product. Therefore, physical parameters such as hardness, stiffness, or transparency easily accessed by the end customer are not so often considered in industrial practice, since they are the result of a series of treatments that the material undergoes before it goes to the costumer. The modification of the PLA bioplastic and its blends with engineering polymers can therefore be very complex [36,37], manifesting itself not only in the change in mechanical properties of the final product but also in its processing, price balance, and long-term durability in various operating conditions. Due to its specific properties and relatively easy modification ability, the use of PLA resin with engineering polymers such as nylons, polycarbonates, styrene-based polymers, or other polyesters has been the subject of numerous scientific studies. 

### 1.3. Processability

The issue of processability may relate to several aspects including the ease of processing, but the principle of optimal choice depends primarily on the method of processing [38,39]. In the category of engineering plastics, the most important production technology is injection molding and its variants such as gas-assisted injection molding technology or physical/chemical foaming injection molding [40,41,42]. In most cases, the low viscosity of the PLA polymer melt is required in this field. Because of that, PLA-based blends have quite favorable flow characteristics, which result primarily from the low viscosity of PLA and its relatively low melting point, at around 160 °C. Both features are useful, especially when compared to the processing of amorphous thermoplastics such as PC, PMMA, or ABS. The required melt processing temperatures for these polymers reach 250 °C or even 300 °C, and the main reason for that is their high viscosity at lower temperatures. The addition of PLA results in a reduction in the viscosity of the mixture, which results in the required flow path of the material in the mold and translates into cost reduction and improved mold cavity reproduction. For the other popular processing techniques such as extrusion of profiles or foils, a reduction in the viscosity or the polymer melting point could be also beneficial, but in this case the viscoelastic properties of the polymer, like melt strength, are equally important and usually deteriorate with decreasing viscosity.

**Figure 2:**
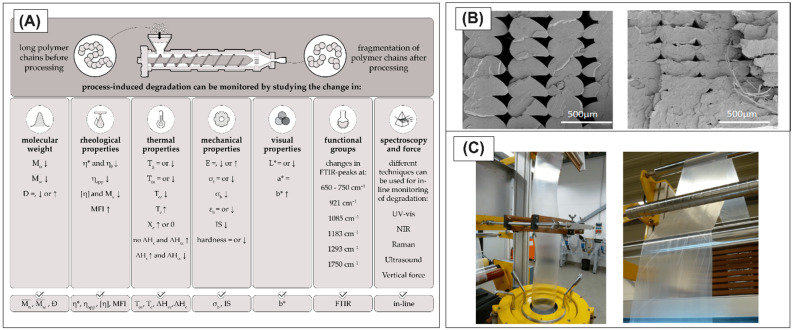
(**A**) A scheme presenting the influence of PLA processing and degradation phenomenon on different properties like molecular weight, viscosity, thermal/mechanical properties, etc. [50]. (**B**) The differences in the filament layer structure of 3D printed parts due to changing viscosity factor [51]. (**C**) Flow instabilities during the foil blown extrusion process, for PLA/PBAT blends [49]. Copyright 2024 MDPI.

Unlike the injection molding method, most of the extrusion-based techniques, foil/film manufacturing, or MEX 3D printing require the use of materials with a relatively low flow coefficient in order to control the thickness or width of the material stream flowing out of the nozzle or die-head shaping the finished product. In such cases, the use of materials with low viscosity could led to a reduction in the melt strength, and as a result, the material spills in an uncontrolled manner [43,44,45]. For the discussed cases, the apparent benefits resulting from using the PLA component in the blend become problematic, as they cause the loss of process stability under standard conditions. For most cases, it is possible to easily optimize the processing parameters, like for extrusion 3D printing [46,47]. However, for large-scale manufacturing techniques like cast/blown film extrusion, it is a time- and material-consuming process [48,49]. The issue of processability of PLA-based polymer systems can, therefore, be a complex problem. Depending on the processing technique and the intentions regarding the modification of the polymer mixture/blend, it can lead to significant changes in the processability of the material (see Figure 2).

### 1.4. Heat Resistance

Like many popular biobased polyesters, PLA also falls into the category of materials with relatively low thermal resistance, posing a significant challenge for potential engineering applications. The primary issue with utilizing PLA in such contexts lies in its slow crystallization process and low glass transition temperature [52,53]. While these properties offer distinct advantages in film processing or packaging production, such as high transparency and reduced processing costs, they often fall short in technical applications where injection-molded parts are predominant. Here, the high content of the crystalline phase typically determines increased thermal resistance, a criterion PLA fails to meet in standard processes.

In traditional blending approaches, addressing this shortfall necessitates the use of a higher proportion of a second, more thermally resistant component. This secondary polymer should either exhibit a higher glass transition temperature, such as PC or ABS [54,55,56,57,58], or possess a notably high crystalline phase content, as seen in polyamides [59,60] or polyoxymethylene (POM). Another approach to improving PLA’s heat resistance is to incorporate rigid segments into the polymer. A successful example is high glass transition PLA, prepared by the ring opening of a cycloadduct derived from lactide [61]. Introducing cyclopentadiene into lactide produces a bifunctional lactide derivative, resulting in a copolymer with a significantly higher glass transition temperature of approximately 190 °C, compared to the typical ~60 °C for standard PLA [62].

**Figure 3:**
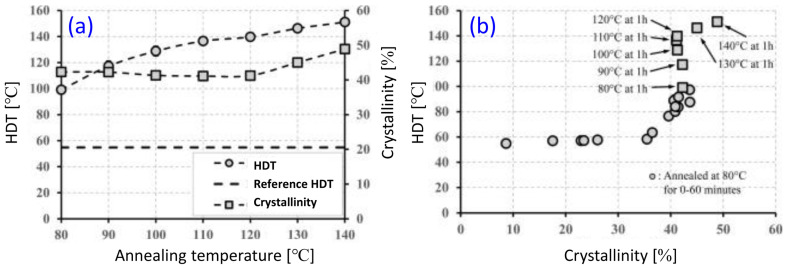
(**a**) Heat deflection temperature (HDT) of PLA annealed at various temperatures for one hour. The dashed horizontal line represents the HDT of the reference (unannealed) PLA. (**b**) HDT of PLA annealed at 80 °C as a function of crystallinity, annealed for various amount of time, and at various temperatures from 80 °C to 140 °C for 1 h. Figure reproduced with permission from Ref. [63]. Copyright 2016 Elsevier.

A more effective strategy to retain the biobased PLA content in the final blend involves modifying the PLA structure to enhance its crystallinity through an annealing process at various temperatures (Figure 3a). In addition to crystallinity, the crystalline form (α′ and α) also affects PLA properties. Controlling the crystalline form can significantly increase the heat deflection temperature (HDT) of PLA. For instance, annealing PLA at 80 °C for different durations to develop various crystalline ratios showed that up to 35% crystallinity, there was only a minor improvement in HDT from 55 °C to 58 °C. However, above 35% up to the maximum crystallinity level of 44%, the HDT increased linearly from 58 °C to 97 °C. Furthermore, when the effect of annealing temperature (80–140 °C for 1 h) was studied, it was found that despite reaching practically the same crystallinity, the HDT could be further improved from 97 °C to 151 °C (Figure 2B). This improvement is attributed to the gradual change in crystal structure from the less ordered α′ to the more ordered α crystal form [63].

Prior research has primarily achieved this objective through annealing the material structure or nucleating the crystalline structure from the melt. However, both approaches incur additional costs associated with the procurement of nucleating agents or the energy required for annealing/heating. Nonetheless, past studies underscore the necessity of conducting such treatments, particularly within the context of potential applications in the machinery or automotive industries. 

In industrial practice, the most popular solution to achieve a high heat resistance index is the use of hybrid modifications, where, in addition to the use of nucleating agents, PLA is additionally mixed with a polymer of higher crystallinity or with a reinforcing filler [32,64,65,66,67]. Due to the efficiency of injection or extrusion techniques, the annealing process is rarely used, which in turn is often used as post-processing in 3D printing [68,69,70].

### 1.5. Toughness and Impact Resistance

Due to its high modulus and strength in both flexural and tensile tests, PLA could be perfectly fitted into the engineering polymer category. Unfortunately, very low impact resistance excludes it from this group. Attempts to improve flexibility and impact resistance are some of the main directions for PLA research, which has resulted in several possible solutions to this problem. Most of the work in this area is dominated by two main directions for solving the brittleness of PLA. The first strategy utilizes the plasticization phenomenon by the addition of low molecular weight compounds [71,72,73,74,75,76,77,78,79,80], which is similar to the case of PVC modification. Some of the plasticizers are partially and fully biobased, which in the context of applications in PLA modification is a significant advantage [81,82,83]. Despite the simplicity of this approach, the use of plasticizers has a number of drawbacks, such as the volatility of plasticizers under processing conditions and their tendency to migrate in the finished product [84,85,86]. The second popular method involves the use of mixtures of PLA with elastomers and the formation of an immiscible blend structure that hinders the propagation of cracks in the material. The effectiveness of this modification method depends largely on the morphology of the resulting phase structure as well as the quality of the interphase surface. These features are influenced by the materials used and processing factors, and the description of their obtained impact strength is the subject of interest of many research articles [87,88,89,90,91,92,93,94,95,96]. 

The use of melt blending modification is the one of most accessible forms of processes for improving the impact strength/toughness of polymer materials, but the use of composite additives can be also an effective method for impact strength improvement. In the modification of PLA-based materials, even the use of standard glass fibers effectively improves the impact strength, while also natural fibers can be effectively used [97,98,99]. The key factor in many cases is obtaining composites with fiber lengths exceeding the standard dimensions of short chopped fibers. The use of long composite fibers is a technological challenge that is solved in the standard injection molding process by using the granulate co-extrusion technique, often called LFT (long fiber thermoplastic) [100,101,102]. In the case of long fiber composite shaping, the most effective solution is to use materials reinforced with continuous fibers, as in the case of composite laminate production, which, however, significantly limits the possibilities of producing complex product geometries. A certain solution in this case is the continuous fiber printing technique, where PLA as the base material used in printing is often subject to modification [103,104,105]. This technique, apart from the obvious increase in stiffness, offers a very significant increase in impact strength that is essentially unattainable for materials manufactured from materials based on PLA blends. Another concept allowing the use of long fibers to improve mechanical characteristics is the laminate overmolding technique, where a reinforcing element made of a fabric-reinforced laminate is placed in the injection mold cavity; this technique is currently gaining popularity, particularly in the automotive industry [106,107]. Despite their high efficiency, long fiber processing techniques require the use of specialized equipment, which is not cost-effective in the case of most popular consumer products. Therefore, from an application perspective, the most practical way to improve the impact strength of PLA-based materials is to use polymer blends or composite systems with the addition of impact modifiers.

### 1.6. Biodegradation and Durability

**Figure 4:**
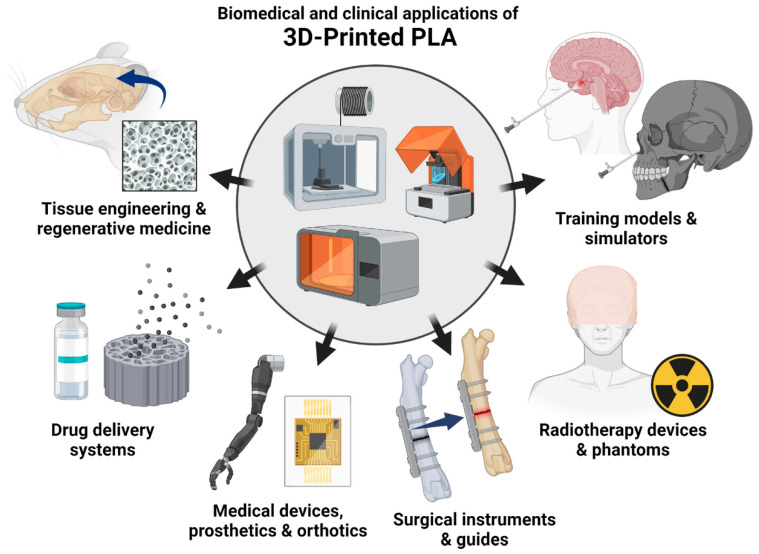
The areas of biomedical applications of PLA-based materials, including non-resorbable usage and tissue engineering using bioresorbable materials. Adapted from Ref. [117]. Copyright 2024 MDPI.

The accelerated degradation process is one of the key features of PLA-based materials; in fact, it is mainly the properties related to PLA biodegradation processes that constitute the main application factor for many industrial areas. Currently, PLA and its derivatives are widely used in the medical industry (see Figure 4), as a material for surgical threads, screws, fixing pins, and bone implants [26,108,109]. In the case of most of these applications, the degradation process of the PLA structure occurs inside the human body, which is why the materials used are often marked as bioresorbable [23,110,111]. Bioresorbable materials are an alternative to titanium and its alloys, which are standard in many areas of surgery but do not always work well as a material replacing the living tissue. This is particularly true for regenerative procedures in which the patient’s bone is not mechanically loaded, or when the area of regenerated tissue does not require permanent stabilization. In ideal conditions, bioresorbable products should be gradually removed from the implantation site by a slow hydrolysis process, so as to allow for the gradual growth of living tissue in the defect or fixation site [112,113,114]. Unfortunately, despite decades of clinical research, bioresorbable materials used to date do not provide a perfect solution. The resorption process, as well as the remineralization of bone tissue, sometimes proceeds in an unplanned manner, which causes post-procedure complications or the need for another surgery. Hence, further work on the development of PLA-based materials in this field focuses on the use of new varieties of mineral fillers, similar to calcium phosphates naturally occurring in bones. The changes also concern the composition of the polymer matrix itself, where currently numerous works are being carried out on obtaining PLA copolymers based on the L-lactide and D-lactide varieties, as well as glycolide and caprolactone [21,115,116]. It is worth emphasizing that unlike technical/engineering applications, where the mechanical properties determine the efficiency of the materials, biomedical applications do not have to meet strict requirements, which is related to the main goal of PLA usage regarding bioresorption processes.

Degradation in composting conditions is the second important reason for using PLA, this time in relation to the packaging industry. The mechanism of PLA degradation in composting conditions is very similar to the bioresorption processes occurring in living organisms. However, in industrial conditions, its kinetics are many times faster, which is mainly due to the high temperature of biomass decomposition processes, often exceeding 70 °C [118,119,120,121,122]. Unfortunately, the high temperature of the composting process is one of the required conditions for effective degradation of the PLA structure, which is why most of the scientific and industrial work carried out in this context concerns attempts to use polymer mixtures with the lowest possible required decomposition temperature, so as to enable composting of packaging under home conditions [123,124,125,126]. Despite the great attention paid to increasing the efficiency of degradation processes of packaging materials based on PLA and its blends, no less attention is paid to obtaining materials with optimized functional properties. For the packaging industry, in addition to the required mechanical performance, the transparency and barrier properties of packaging are also of great importance [127,128,129,130,131].

Because the primary advantage of PLA is its ability to fully decompose under composting conditions, its long-term durability stays in opposition to its basic feature as a biodegradable polymer. However, in the case of engineering applications, this problem should find a possible solution if PLA-based mixtures are going to find a wider application. Previous studies in this field confirm the short-term stability of the blend properties. The main cause of the decrease in strength characteristics is the predominantly rapid degradation of the PLA structure, particularly intense at elevated temperatures and in the presence of water. This process has a strong negative effect on the quality of materials even with a low PLA share in the structure, mostly due to the negative impact of PLA degradation products on the durability of the remaining components of the mixtures and the interphase between them, a phenomena which was reported for blending PLA with such polymers like PC [132,133] and PMMA [134,135]. 

### 1.7. Price and Eco-Balance

The high price of biopolymers is still a major problem that determines their limited use. According to actual market trends (2023–2024), the price for industrial-grade PLA varies from 2.6 to 3.3 USD/kg (Procurement Resource, 2023 report). The purchase price for most popular thermoplastics such as polypropylene (PP), polyethylene (PE), or polyethylene terephthalate (PET) rarely exceeds the level of 1 USD/kg, which is still a huge difference, causing a low demand for biopolymers. It is worth mentioning, however, that PLA’s position in the large-volume plastics market may strengthen over the next few years due to the constantly growing demand for this polymer. Currently, it is widely used in several niche markets, but in the growing demand for biobased products, the PLA material market position may only grow.

In the biomedical industry, where the cost of certified bioresorbable materials can reach several hundred euros per kilogram, such a high price is not an obstacle because it is comparable to or much lower than competitive titanium alloys. However, considering the low-tonnage consumption of medical PLA varieties, this industry does not constitute a promising market for the development of the biopolymer concept. 

A much more promising way of developing the biopolymer market, including PLA and its mixtures, is the packaging industry, where the growing demand for foils/films capable of decomposing in composting conditions may contribute to an increase in the production of biobased/biodegradable polymer materials. At present, the most common PLA-based materials are mixtures intended for the production of thin films by the blowing extrusion method, where, in addition to the PLA component, the material contains such biopolymers as polybutylene adipate terephthalate (PBAT), polycaprolactone (PCL), and polybutylene succinate (PBS) [136,137,138,139,140]. Unfortunately, despite the possibility of synthesis of these polymers from renewable raw materials, the production methods still dominant in the industry are based on petroleum derivatives. The obvious problem is still the high price of all of the polymer components of the mixture, where PLA is often the cheapest component. An intermediate solution to the problems in reducing the price of biopolymer mixtures is the use of thermoplastic starch. With appropriate optimization of the composition, biodegradable mixtures can reach up to 50% TPS, which significantly contributes to a reduction in the material price [19,141,142,143].

An interesting example of a technology where the cost of PLA-based blends is not an obstacle to its application is the material extrusion 3D printing technique (MEX). In most cases, PLA filament is the cheapest alternative to other polymers such as PETG, PCTG, and HIPS. Despite the lower cost of the raw material, the price for PETG/HIPS-based filament is higher than that of PLA. The reasons for this trend should be sought in the development of the filament production market itself, where the share of PLA has been over 50% of production for several years (according to Filamentive Limited). It is worth pointing out, however, that as in the case of the biomedical industry, the unit cost of purchasing a kilogram of filament significantly exceeds 10 Euro, which is several times the purchase price of the pure raw material. 

The price difference between the higher cost of purchasing engineering plastics and the generally cheaper commodity polymers does not reflect the overall distance in the cost of manufacturing for the mass production of goods and technical products. The unit cost of engineering plastics products is mainly raised by the higher price of tools/machines and the increase in energy consumption associated with specific processing parameters like higher temperature and pressure. The other factors that increase the price gap are so-called peri-process activities such as drying, machining, or conditioning. Therefore, despite of the higher unit price of PLA in comparison to commodity plastics like PP, PE, or PS, the PLA-based engineering purpose blends are attractive not only because of the lower price of the prepared pellets, but primarily because of easier processing of such blends. 

In terms of ecological value, the idea of using PLA/engineering polymer blends does not make it possible to identify such materials as biodegradable or compostable, which is often overused by many manufacturers and researchers. The key to the use of this type of materials is the principle of renewability of raw resources. The ability to reduce the consumption of petroleum-based products helps to improve the carbon footprint of this type of product, which is the basis for most of international agreements on reducing the global effects of climate change since the Kyoto protocol in 1997.

This article provides an overview of current endeavors utilizing PLA and its modification for engineering applications, detailing the material design and methods employed to achieve desired engineering properties through microstructural analysis, while exploring potential avenues for future development. 

## 2. Methods of Modification of PLA

### 2.1. Increasing of the Toughness and Elasticity

For most thermoplastic polymers, modification to increase the toughness or impact resistance is a key step that manufacturers of engineering plastics must consider during processing. In current research practice, two main methods of PLA impact resistance modification have been adopted: mixing with plasticizers, or another, more flexible polymer. In both cases, the modification takes place during melt processing, but due to different mechanisms of interaction, these processes represent the different areas of modification often with opposing effects and objectives. Therefore, the effectiveness and popularity of each of them depends on many factors and, above all, on the intended use. In the case of brittle engineering plastics processing, a greater popularity is achieved by blending with more ductile polymers/elastomers, mainly due to a higher efficiency as well as a lower impact on other crucial properties of the base polymer. This does not prejudge the interest of researchers in the field of plasticizing techniques [144].

For all construction materials, two main types of fracture behavior can be observed and named as brittle and ductile. For the brittle type, the plane of the fracture is perpendicular to the applied tensile stress, and the material is not subjected to significant deformation. In turn, the ductile type is characterized by considerable plastic deformation. In the case of engineering materials, the more desirable is the ductile/plastic deformation behavior type, mainly due to the higher energy required for this destructive deformation, as well as the slower, and thus more predictable nature of this process. In the case of polymer-based materials, two basic factors determining the brittle or ductile fracture behavior are the temperature and speed of the deformation. In the case of relatively low temperatures, of which the upper limit is determined by the glass transition temperature, the change in the atomic structure (i.e., the conformation of the polymer chains) is limited by their lack of mobility. Thus, for high molecular weight polymers, the entanglements of the chain structure hinder their mutual slip while the deformation rate reaches the critical value and causes the mechanical dissociation of the chemical bond of the polymer molecule. The brittle fracture of the sample is the result of a progressive burst reaction of the material bonds, initiated by a single chain break which has caused the transfer of the load to the surrounding chains. As mentioned before, the other factor that induces such behavior is increasing the speed of deformation, where even in the material’s elastic region (above the glass transition) brittle behavior can be observed. This is due to there being insufficient time for a possible rearrangement of the structure, as the result of a very high propagation of stress, which happens during impact tests. 

During real observations, we can distinguish two main types of micromechanisms of destruction in polymers: crazing and shear yielding, or a combination of both. Most often they occur simultaneously, and the dominance of one depends on many factors, such as the polymer phase structure, surrounding thermal conditions, and deformation rate. Since most of the engineering polymers are semicrystalline materials, additives and fillers are also present in them, and therefore the additional micromechanisms affecting the fracture behavior of polymers are related to the formed phase interfaces. The two most important of these are cavitation and de-bonding, and like others, are also dependent on deformation conditions, which can accelerate or reduce the tendency of brittle fracture.

For PLA-based blends, the main reason for the lack of fracture toughness is the low impact resistance of PLA compounds dominated by the crazing mechanism, which results in the poor toughness of the entire blend. The research direction in the field of PLA toughness enhancement could also lead to the improvement in the blend properties. It is relatively simpler to improve tensile toughness than to enhance impact toughness in PLA blends. Two main toughness improvement strategies are based on the use of plasticizers and a dispersed elastomeric phase. 

#### 2.1.1. Plasticizers

PLA is a relatively brittle polymer. By the incorporation of plasticizers, which are typically low molecular weight compounds, the flexibility and ductility of PLA can be increased. This increase in flexibility helps to improve the toughness of the PLA, making it less prone to cracking and more resistant to impact. Many low molecular weight compounds, like polyadipates [145], citrate esters [146,147], epoxidized oils [148,149], lactic acid oligomers [150], polyethylene glycol [151,152], and more have been used as plasticizers in PLA formulations. Most plasticizers used in the market are made from petrochemicals and synthetic materials. Biobased, green plasticizers derived from renewable and biodegradable resources have recently attracted significant interest for PLA use. Some examples include cardanol oil [153], egg yolk oil [154], Anatolian sweetgum oil [155], TEC, and glycerol triacetate (GTA) [156]. However, the selection and concentration of the plasticizer are crucial, as excessive plasticizer content can lead to a decrease in mechanical strength and modulus and may also affect other properties such as thermal stability and biodegradability. Additionally, the choice of plasticizer should also consider the intended application and any specific requirements, such as food contact or biocompatibility.

The plasticizing phenomenon, in the most general terms, leads to an increase in the distance between polymeric chains, which in turn results in a reduction in physical interactions between the chains of the modified polymer and increases their mobility. The insertion of plasticizers between the longer base polymer chains causes the intermolecular space to increase. Monomers or low molecular weight compounds that are miscible with the base polymer could be used as plasticizers. The plasticization mechanism is used primarily as a processing modification, since the slip between polymeric chains effectively lowers the viscosity. Because of the typically physical nature, the effectiveness of the plasticization method is apparent only when the content of the plasticizing compound is relatively high, usually from 20% to even 60%. In the case of PLA modification and its blends, the addition of plasticizers and increased mobility of the resulting polymer structure induces several structural changes. 

**Figure 5:**
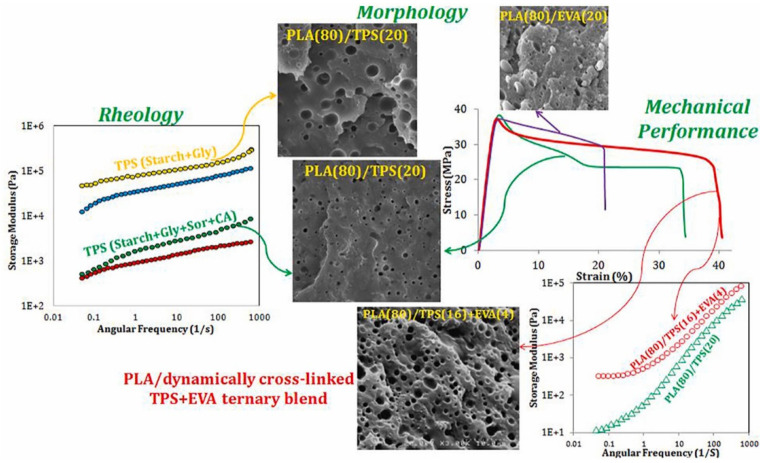
Morphology development and improvement in mechanical properties of PLA with plasticized starch (TPS) binary blends in comparison with PLA/dynamically crosslinked “TPS + EVA” ternary blends. Figure adapted with permission from Ref. [157]. Copyright 2022 Elsevier.

Melt viscoelasticity properties can provide important information about how plasticizers affect microstructure development. The addition of citric acid significantly improved the processability of a thermoplastic starch (TPS)/PLA blend, aiding in the breakup of TPS droplets during melt blending with PLA. This led to a substantial reduction in TPS particle size in PLA/TPS (80/20) blends [157]. Furthermore, blending dynamically cross-linked TPS with ethylene-vinyl acetate (EVA) and PLA resulted in a PLA (80 or 60 wt%)/TPS + EVA ternary blend with a unique matrix-dispersion morphology. The resulting morphology significantly enhanced the toughness of the ternary blend (PLA/TPS/EVA) compared to PLA/TPS binary blends (Figure 5).

Changes during the fracture process, in the case of modification of polymers by plasticizers, are mainly related to the decrease in the glass transition temperature. PLA exhibits a relatively high glass transition temperature of ~60 °C. The introduction of small molecules increases the distance between individual polymeric chains. The subsequent increase in distance results in a decrease in interactions, so the stresses in a single polymer chain are not so readily transferred to the other molecules. The increase in distance also causes a decrease in the level of entanglements in the polymer structure, which facilitates mutual slippage and rearrangement of the chain network during deformation. These changes obviously depend on the content of the plasticizer in the polymer structure; at high content levels, causing a complete change in the nature of the polymer from rigid and brittle to flexible and ductile leads to an improvement in its fracture toughness. 

Plasticization with monomers and low molecular weight compounds is a known and practiced process for many thermoplastic polymers. The benefits of using this method are mainly due to the high degree of miscibility of low molecular weight particles. In the case of previous research on PLA plasticization methods, a wide range of chemical compounds has been used. One of the most effective PLA plasticizers is the lactide monomer, which is quite understandable considering other polymers where plasticization is carried out in a similar manner. However, the disadvantage of using low molecular weight compounds is their low stability as a function of time. This is evident in their tendency to migrate towards the surface of the product, and the effect of this phenomenon is a progressive decrease in the mobility of the polymeric chains and therefore also a drop in the impact resistance value. The general trend for all plasticizers is the causal link between the decreasing molecular weight of the modifier and its growing tendency for efficient plasticization, associated with miscibility with the base polymer. The same but opposite trend applies to unfavorable phenomena, which is the migration of the plasticizer particles to the surface and their evaporation during processing. With the increasing size of the plasticizer particle, their tendency towards these unfavorable phenomena decreases. Furthermore, plasticization of PLA or PLA-based blends could reduce the HDT of the material considerably [158].

Most of the research studies dealing with the subject of plasticization in the case of PLA are focused on the optimization of the composition towards achieving the best balance of impact, optical, and thermomechanical properties, while at the same time improving the plasticization effect, reducing evaporation at the processing stage, and reducing migration towards the surface. The results are mainly applicable in the case of the packaging industry, where for many reasons the required feature is the material transparency and related amorphous structure domination [155]. In the case of engineering applications, this requirement is not so common and, in many cases, impossible to fulfill by semi-crystalline polymers such as PLA.

#### 2.1.2. Elastomer Modifiers

The most used method of improving the impact resistance of polymers is the use of elastomeric modified blending. The most popular example of this type of process is the use of polypropylene (PP) modified with EPDM [159,160], intensively used during the production of car bumpers or electrical cable connectors. The modification of this type was dictated not only by the operational requirements, but also the need to use the cheapest possible solution, due to the large-scale nature of production. Unlike the plasticization phenomenon of polymers described quite well in the literature, especially in the context of PVC [161,162,163], the modification and mechanism of interactions in polymer/rubber blends is still an open subject of the discourse of chemists and physicists, mainly due to the complex arrangement of many competing processes occurring in polymers. The fracture behavior during the impact tests is also difficult to observe and simulate. 

**Figure 6:**
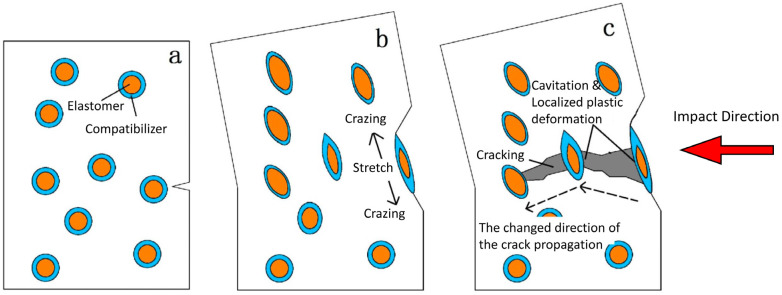
Phenomena of deformation and toughening mechanism of elastomer in polymer upon impact strikes. (**a**) Dispersed elastomeric phase, before deformation; (**b**) low strain structure deformation, before initiation of the crazing/cracking mechanism; (**c**) high strain structure deformation, intensive crack propagation, cavitation of the elastomeric phase inclusions Modified and reproduced from Ref. [164]. Copyright 2018 MDPI.

Figure 6 describes the phenomena of deformation occurring in impact-modified polymers, and points to several main reasons for the increase in the impact toughness, as follows:

Termination of the spreading of microcracks on the surface of the elastomer phase, which prevents cracks from spreading across the entire volume of the material (Figure 6b).

Formation of crazes around the elastomer particles, resulting in energy dissipation increasing with better energy dissipation, which also eliminates the concentration of stress (Figure 6b).

Cavitation of the elastomer particles under stress, leading to a reduction in hydrostatic stress at the top of the microcrack and enabling local plastic deformation to absorb the impact energy (Figure 6c).

The intensity of the occurrence of each phenomenon is largely dependent on the size and dispersion of the elastomer phase, and the strength of the polymer/elastomer interface.

Blending PLA with elastomers or ductile polymers has become a straightforward and effective method for enhancing its toughness. However, merely blending with rubbers may limit the extent of toughness improvement. Further innovation in processing methods and phase compatibilization is necessary to tailor the toughness of these blends to achieve a super-tough performance. Unlike PLA alone, blends of 80/20 PLA/ENR exhibit a significant improvement in notched impact strength. Moreover, the impact strength was further enhanced by 180% with the addition of an optimal amount of nanosized silica [165]. The role of nanoparticles in enhancing PLA’s toughness involves several mechanisms, such as reinforcement, barrier effect, interface interaction, etc. The synergistic enhancement of the strength and toughness of the PLA blend is achieved by leveraging the selective dispersion effect and manipulating the compatibility and phase structure of blends with the presence of nanoparticles in the system [36]. Incorporating nanofillers such as nanocellulose, nanoclay, carbon nanotubes, and functional nanomaterials allows for the precise tuning of PLA properties for desired applications [166].

The final toughness value of PLA/rubber blends is influenced by various factors, including the type and quantity of rubbers used, the shape, size, and distribution of rubber particles, as well as the interparticle distance within the dispersed phase. Additionally, the interfacial adhesion rubber network linkage and phase structure of the blends play crucial roles in determining their toughness. 

**Figure 7:**
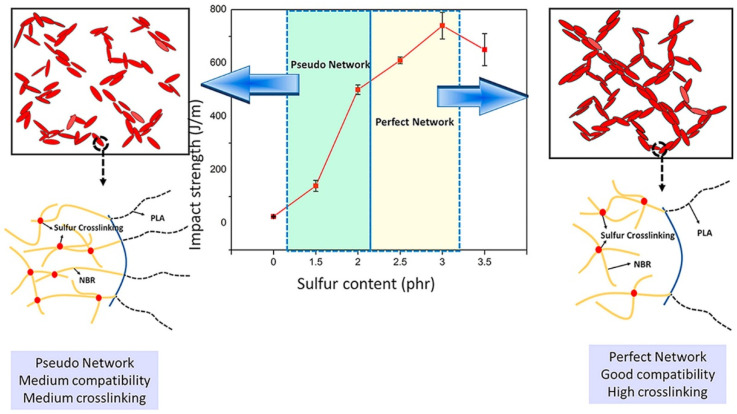
Toughening polylactide by dynamic vulcanization with NBR through morphology transformation from pseudo to perfect rubber network with optimized sulfur content. Figure adapted with permission from Ref. [172]. Copyright 2021 Elsevier.

Dynamic vulcanization, which involves selective crosslinking of a rubbery polymer during melt blending with a thermoplastic, has proven to be a highly efficient method for toughening brittle plastics. Many super-tough PLA blends have been prepared using this technique [167,168,169,170,171]. Super-tough PLA/acrylonitrile butadiene rubber (NBR) blends were created using a dynamic vulcanization process [172]. The sulfur content was crucial in transforming the blend morphology and impacting the final impact strength. Figure 7 shows that the optimal sulfur content significantly enhances impact strength by forming a strong rubber network and improving interfacial adhesion.

Toughening polymers with a rubber phase can have one drawback, i.e., a drastic reduction in mechanical strength and modulus. However, even with the addition of rubber to PLA, which slightly reduces these properties, PLA still maintains satisfactory overall mechanical performance due to its inherently high tensile and flexural strength, as well as its high tensile and flexural modulus. Various flexible and elastic sustainable polymers, whether synthetic or biobased, have been used. However, biobased elastomers or high-toughness biobased polymers are ideal choices to enhance the toughness of brittle PLA without largely compromising its biodegradability or sustainability. Some of the popular high-toughness biobased polymers include PBAT, PBS, and PA11. Blending PLA with biobased polymers such as PBAT has tremendous potential to replace the widely used non-biodegradable plastics across various applications, especially in the packaging sector. Increased research in this field can accelerate the adoption of more eco-friendly PLA blends by enhancing their properties and reducing costs [173,174].

**Figure 8:**
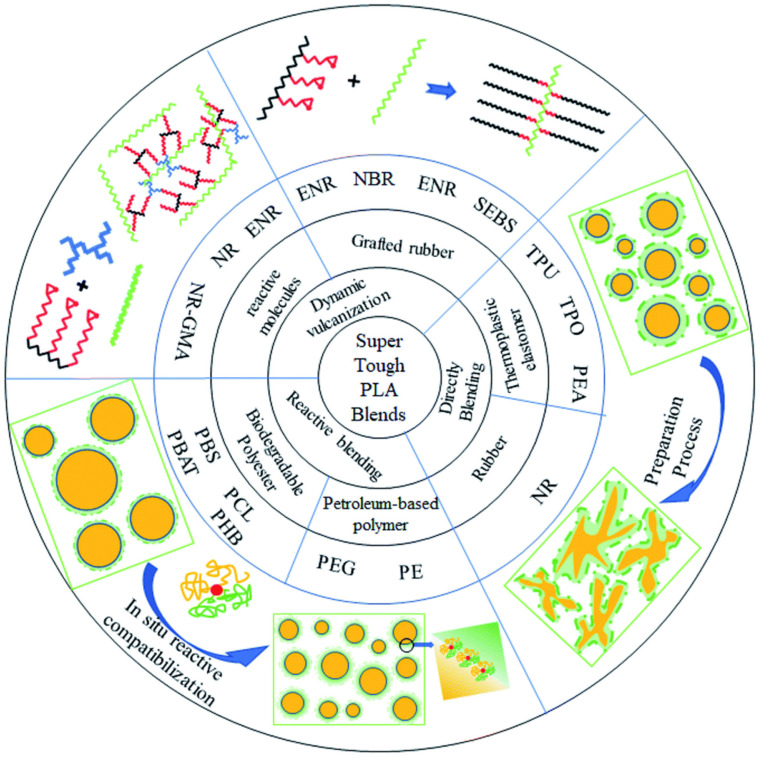
The summarized approaches and toughening strategies for enhancing the toughness of PLA-based blends. Adapted with permission from Ref. [36]. Copyright 2020 RSC.

Figure 8 presents the simplified processing strategies and approaches for enhancing PLA toughness using various types of elastomers and biobased polymers to achieve super-tough performance.

Many comprehensive reviews on toughening PLA with various types of elastomers and reactive elastomeric materials have been published and are readily available [36,37,175,176,177]. Therefore, we did not delve into detailed reviews of these aspects in this article. 

### 2.2. Heat Resistance Increasing

The high temperature resistance of polymer-based materials, as well as other thermomechanical features, is closely related to the behavior of the macromolecular structures of polymers. As with other features, the important factor is the glass transition region of the specific polymer and its ability to create the ordered crystalline structure. In simple terms, the deformation of the material by applying a relatively low stress is most likely due to the mobility of the polymer chains in the polymer structure. In the case of amorphous polymers, the possibility of structural rearrangement is closely related to the temperature range of the glass transition, where the increasing mobility of the structure of individual polymeric chains facilitates their mutual slip and the deformation of the material. In the case of semi-crystalline polymers, the contribution of the strong ordered phase results in a change in behavior towards the amorphous plastics. The glass transition and associated weakening of intermolecular interactions are closely related to the amorphous phase, while the crystalline phase transition occurs at the melting temperature. Because the disintegration of the crystalline structure requires more energy, the melting point therefore occurs at higher temperatures. The result of that is the increase in the deformation resistance and is strictly dependent on the crystalline content in polymer structures.

The structure of the polymer chain, its length, branching, and other factors determine the nature of the formed crystalline phase. Therefore, the formation of the ordered structures in each polymer also depends on thermodynamic factors such as the process temperature gradient or the cooling rate. In practice, they are the decisive factor in the growth dynamics of the crystalline phase. Due to their thermomechanical properties, including deformation resistance at high temperatures, highly crystalline polymers are preferred. For most of the commonly used engineering plastics, the formation of the crystalline structure does not require any additional treatment. The crystalline phase growth rate for polymers such as PA, POM, or PBT is high enough that any changes in the injection molding parameters does not significantly affect the intensity of its growth. Unfortunately, for some thermoplastics polyesters, including most popular PET and biobased PLA, the crystalline phase growth is very slow. The increase in the crystalline phase would require significant changes in the molding process conditions, leading to high temperatures and a long period of time, which would be economically unreasonable.

For both polymers, due to the possible benefits, the crystallization process can be accelerated or intensified. Because of the many similarities, processes that have been implemented so far with PET are also reflected in the modification of PLA, which is applied to the modification to increase the crystalline phase share in the polymer. Like PET, PLA is also susceptible to annealing, and after heating and exceeding the glass transition temperature undergoes the cold crystallization process, which effectively increases the crystalline phase content. Another effective treatment is the crystalline phase nucleation during the cooling stage of the molding process, which has so far been successfully used for PET and could also be efficient for PLA. The principle of both processes is quite different. However, in both cases, the aim of the treatment is to increase the crystalline phase, which in turn results in increasing deformation resistance at high temperatures.

#### 2.2.1. Annealing

In addition to basic molding processes such as extrusion or injection molding, there are also many post-processing treatments such as painting, welding, and machining. In selected cases, heat treatment is also used. The most common example here is the conditioning and annealing of polyamide-based parts. In the case of conditioning processes, the purpose of the treatment is to increase the flexibility of the molded parts, for which the corresponding water content in the structure results in a plasticization effect. In the case of annealing, the purpose of the treatment is mainly connected with removing the stresses which are generated during the molding stage. In both cases, the treatments are carried out using a furnace or chamber with controlled temperature and humidity. The treatment time depends mainly on the wall thickness of the parts and may take anywhere from several hours to even a few days. The polyamide example confirms the need and effectiveness of this type of post-process modification. For PLA-based mixtures, the annealing process seems to be one possible road to improving the selected properties, which could consequently extend the possible applications.

In the case of annealing PLA and PLA-based blends, the main purpose of this operation is to increase the content of the crystalline phase, which in consequence should significantly improve the range of operating temperatures and properties of PLA-based materials. Crystalline morphologies and phase structures play a decisive role in determining the properties of polymer blends. The detailed description of the annealing effects on the properties of pure PLA materials has been described in several publications [178,179,180,181]. This applies not only to injection-molded parts, but also to PLA-based films [182] and PLA-based blends [183]. The phenomenon of annealing is particularly important in the case of processing films, especially for oriented semi-crystalline thin foils made from PET, but this fact does not exclude the imposition of the same mechanisms for PLA. Annealing of oriented films is then carried out simultaneously with stretching above the glass transition temperature. The increase in crystallinity during the annealing/stretching process is highly important for films with controlled mechanical and thermal properties, while maintaining transparency [184,185]. However, in the case of polymer blends, the main research topic focuses on the effect of annealing on the mechanical properties of prepared materials, in particular on impact strength [183,186]. In some cases, marginal improvement in the notched impact strength of PLA blends was observed after annealing [187]. Adding lysine triisocyanate (LTI) can enhance the compatibility of PLA and PCL through additional polymerization [183]. This reduces the size of the PCL phase, leading to a higher fracture energy. Annealing PLA/PCL with the presence of LTI further strengthens the microstructure, resulting in a significantly improved fracture energy.

The increase in the temperature resistance is the most noticeable effect of annealing. However, the reconstruction of the phase structure carries all the related consequences, which fortunately in the case of PLA and its blends is most often positive [187]. The improvement in heat deflection temperatures (HDT) of annealed PLA-based blends and their composites has been widely reported and the detailed annealing conditions and the highest HDT achieved are presented in Table 1.

**Figure 9:**
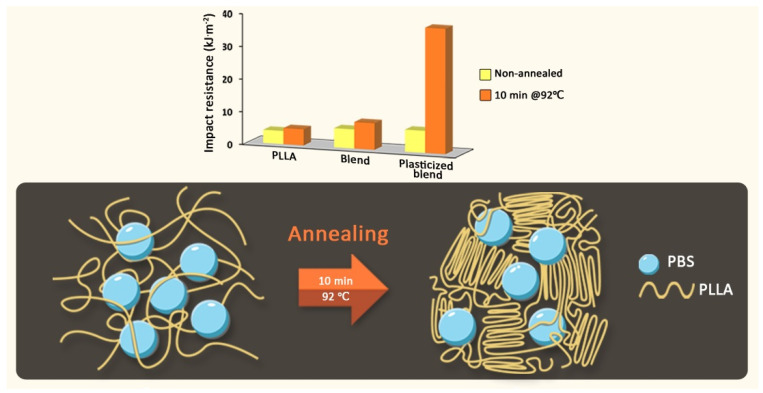
Controlling crystallization and molecular re-arrangement for improvement in impact strength of plasticized poly(L-lactic acid) and poly(butylene succinate) blends. Adapted from Ref. [158]. Copyright 2021 MDPI.

In addition to the natural tendency to increase the stiffness and hardness along with the increase in the amount of the crystalline phase, the impact strength is often improved despite the decrease in elongation at break during the static tensile test. This toughness improvement is mostly related to PLA-based materials being modified with elastomer particles. However, this improvement can be limited with some polymer systems, where increasing crystallinity alone does not ensure the enhancement of impact toughness [158]. For instance, a blend of PLA and poly(butylene succinate) (PBS) increased tensile elongation but had little improvement in its impact resistance. Further adding PEG plasticizer or heat treatment did not achieve the expected improvement in impact resistance properties for the PLA/PBS blend. The impact resistance of PLA/PBS blends increased significantly (~10-fold) when 5% PEG was added as a plasticizer and heat treated at 92 °C for 10 min [158]. The authors attributed these effects to the combined influence of enhanced crystalline perfection and improved compatibility within the system, which was facilitated by PEG rearranging polymer chains into a more orderly manner, effectively acting as a compatibilizer to enhance compatibility between the PLLA and PBS phases in the blends, as shown in Figure 9.

In current industrial practice, the annealing of PLA is not widely used. Despite many advantages, this method is time-consuming and energy-consuming, and there is no greater chance of being popularized. This is mainly due to the large-scale nature of PLA goods production, where in-line modifications are preferable. For this reason, the nucleation method seems to be a more prospective route for increasing the crystallinity of PLA-based materials. 

#### 2.2.2. Nucleation

The necessity of applying nucleation treatment is most often triggered by the need to accelerate the crystallization process or increase the crystalline phase content. The second most important reason is the homogeneity of the crystalline structure throughout the whole molded part. In the case of semi-crystalline polymers, the nucleation process is mainly heterogeneous, while homogenous nucleation is practically absent. The heterogeneous nucleation phenomenon is based on the nucleation of the crystalline phase on the interface surface. Additives, fillers, and even impurities may be the source of this interphase surface. To increase the intensity of the nucleation, it is possible to intentionally introduce the nucleation agents into the polymer matrix. As the result of their introduction, the kinetics of the crystalline phase growth are higher and the crystallization temperature increases. 

Most commonly, the nucleation of the crystalline structure is used to modify polypropylene, the process of which could affect the nucleation of two types of PP crystalline forms, which additionally improves the transparency of the final product and their mechanical properties. In the case of biobased polyesters such as PLA, a very slow crystallization process is usually useful to obtain an amorphous structure and associated high transparency of foils or bottles. In technical applications, especially injection components, slow crystallization is the reason for many limitations. These relate to thermomechanical properties, especially the resistance to deformation at high temperatures, and involve process difficulties involving part demolding and permanent deformation of not crystallized soft elements.

**Figure 10:**
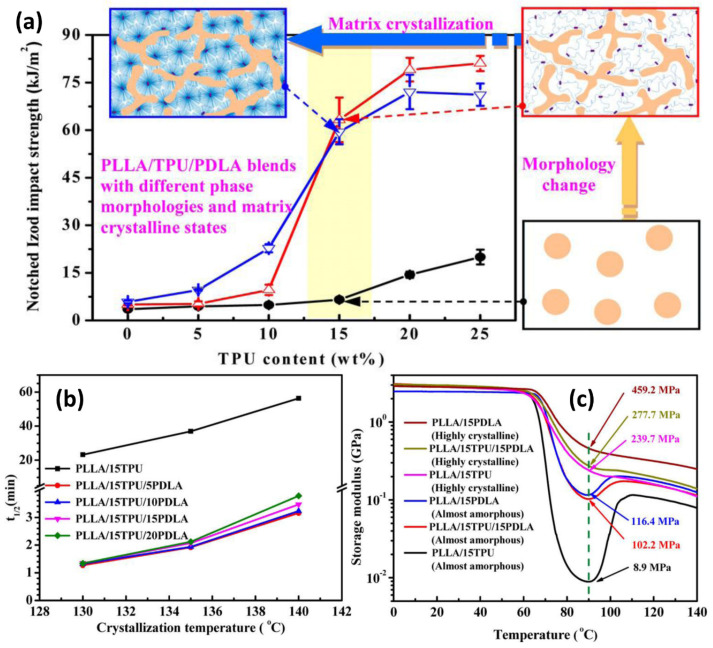
(**a**) The remarkable improvement in impact toughness of PLLA/TPU blends with the addition of PDLA though morphology and phase changes. (**b**) Half-crystallization time (t½) as a function of crystallization temperature for PLLA/TPU (85/15) blends with various contents of PDLA. (**c**) The storage modulus indicating the heat resistance of injection-molded PLLA/15TPU and PLLA/15TPU/15PDLA blends with different PLLA matrix crystalline states. Adapted with permission from Ref. [196]. Copyright 2015 ACS.

The current studies related to PLA nucleation trials have focused primarily on attempts to improve the crystallinity of pure PLA. However, the nucleation of the crystalline phase of PLA-based blends is a topic that has also often been undertaken. For most PLA blends and composites, the effect of increasing heterogeneous nucleation is often noticeable without the intentional use of nucleating agents [60,194,195,196]. For many blends a sufficient factor for crystalline growth is the presence of a highly dispersed biphasic structure, where the interface surface proves to be a sufficient source of nucleation for the crystallites. Liu et al. [196] improved the toughness of PLLA blends by adding small amounts of poly(D-lactide) (PDLA) into thermoplastic polyurethane (TPU)-toughened PLA via melt-blending. The added PDLA chains can easily interact with PLLA matrix chains, quickly forming stereocomplex crystallites. These crystallites act as efficient modifiers, significantly improving the melt viscoelasticity of the PLLA. They also change the structure of the dispersed TPU phase from typical sea–island to a unique network-like structure, enhancing the impact toughness as well as the heat resistance of PLLA/TPU blends (Figure 10a,b). At 90°C, the value of the storage modulus increases significantly from 8.9 to 102.2 MPa. In PLLA/15TPU/15PDLA blends with highly crystalline matrices, heat resistance is further enhanced by the complete crystallization of the matrix (Figure 10c). Additionally, the formed stereocomplex crystallites can speed up matrix crystallization, allowing the preparation of highly crystalline PLLA/TPU blends using standard injection molding.

**Figure 11:**
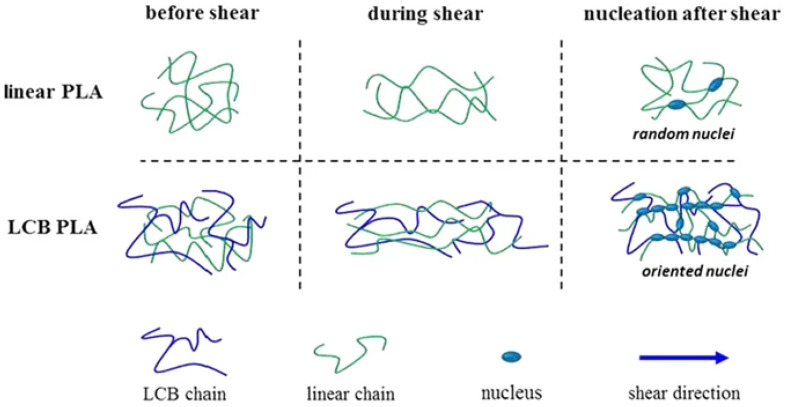
Schematic illustration for the formation of spherulitic and oriented crystalline morphologies for linear PLA and LCB PLAs, respectively. Adapted with permission from Ref. [198]. Copyright 2016 Nature.

By introducing a long-chain branched (LCB) structure into a linear PLA, LCB PLAs crystallize much faster than linear PLA, and this crystallization rate is further enhanced with increasing the LCB degree through increasing the amount of trimethylolpropane triacrylate (TMPTA) under γ-radiation [197]. Crystalline morphologies of LCB PLAs show increased nucleation density with a higher LCB degree, transitioning from spherulitic to oriented crystalline structures [198] (Figure 11). In addition to introducing LCB structures, shear flow is an effective method for enhancing PLA crystallization, which occurs during polymer processing techniques such as extrusion, injection molding, and blow molding [199,200]. Nucleation density significantly increases with shear flow, reaching a saturation point over time under shear. The increase in nucleation ability and the transition from spherulitic to shish-kebab structures induced by shear flow can be attributed to the broader and more complex relaxation behaviors of LCB PLA [199]. Adding a nucleating agent is an effective way to accelerate the crystallization of PLA [201]. In many cases, the use of additional nucleation agents does not lead to an increase in the crystalline phase fraction content, but results in its significant fragmentation/size reduction, hence improving the degree of crystallinity.

**Figure 12:**
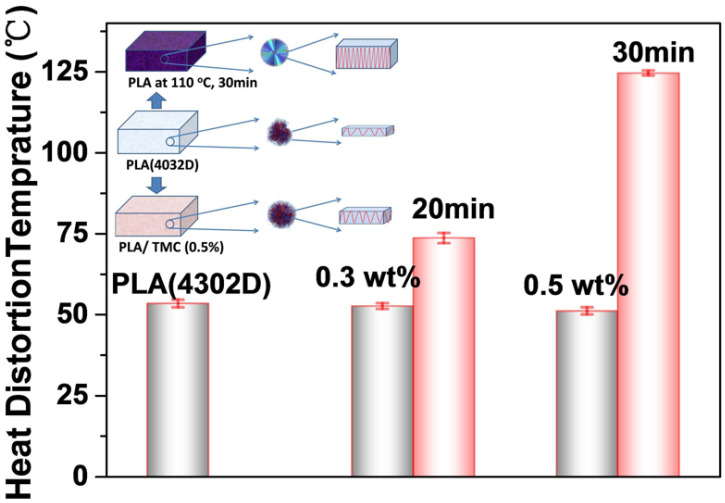
Heat deflection temperature (HDT) of neat PLA, PLA/TMC (0.3 wt% and 0.5 wt%) composites, and PLA treated at 110 °C under varying heat treatment durations (20 and 30 min). Adapted from Ref. [202]. Copyright 2020 MDPI.

Neat PLA has a crystallinity of about 11% and an HDT of around 55 °C. Nucleating agents can increase the crystallinity of PLA but do not improve its crystallization morphology. At the same crystallinity level, PLA undergoing heat treatment can exhibit better crystallization morphology and higher heat resistance when compared to that with the addition of nucleating agents. PLA treated at 110 °C for 30 min has a similar crystallinity to PLA with the addition of a nucleating agent (zinc phenylphosphonate, brand TMC at 0.5 wt%) [202]. However, its HDT is 72 °C higher (Figure 12). This indicates that heat-treated PLA forms a more complete crystal morphology than PLA with the addition of nucleating agents. Therefore, crystallization morphology significantly affects the heat resistance of PLA, and hence the HDT value.

## 3. Commonly Used Engineering Polymers and Their Blends with PLA

Due to the great popularity of blending techniques in industrial practice, the subject of this modification process also applies to important polymers such as PLA. The popularity of this polymer is largely contributed to the fact that as one of the few so-called biopolymers, it is both biobased and biodegradable. These two main reasons have led to the launch of the mass scale production of this polymer, which has made it popular among researchers, polymer processors, and consumers. A pure economic balance would never allow PLA to be so popular, but the growing interest of consumers has forced the petrochemical industry to slowly shift towards a more sustainable production strategy. 

In the case of PLA-based blends, due to existing applications in the packaging industry, the main requirement for the used materials was their biodegradability, which involves using a relatively limited number of possible polymers. The purpose of creating these mixtures is to optimize costs or improve properties of the final product. While the first economical condition is quite obvious in the manufacturer–consumer relationship, the assessment of the property’s improvement direction is already difficult, depending mainly on the operating requirements of the product. In the case of the packaging industry, many mechanical characteristics such as elongation at break, impact strength (tear resistance), and stiffness could not be mentioned as the main directions of modifications. More often the features like transparency, chemical resistance, or gas permeability are taken into consideration. At present, most of these requirements can be fulfilled using PLA blends by the appropriate selection of material composition and processing method. The current subject of intensive research is the use of PLA blends in technical applications, especially injection-molded parts used in the electronics, automotive, and machine industry. Examples of effective combinations of PLA and engineering polymers described in the literature are not yet a breakthrough in this field, but demonstrate the possible use of this type of polymer materials. 

### 3.1. Engineering Polyesters (PET and PBT)

An important factor in the growing popularity of PLA is its relatively good processability. In many cases, the forming processes of PLA are like those used for poly(ethylene terephthalate) (PET) processing. At present, PLA-based resins could successfully replace PET because modern processing lines can effectively work with both polymers without the need to modify the processing machines or tools. The only barrier seems to be the price and properties. For technical applications including injection-molded parts, PET has many uses, due to several features not found in other polymers. These are excellent creep resistance, low coefficient of friction, and scratch resistance. Like most engineering polymers, PET injection molding is a very expensive and demanding processing technique. The main applications of PET resins are the housings of lamps and sensors, electrical connectors, and transformer housings. However, PET has a slow crystallization rate during the melt-crystallization process in comparison with other polyesters such as poly(butylene terephthalate) (PBT) and poly(trimethylene terephthalate) (PTT). 

Despite the quite obvious similarities between PLA and PET in the studies, so far, the blends of these two polymers have not often been investigated. The fundamental obstacle may be a significant difference in the processing temperatures, which makes it difficult to carry out the melt blending process. PLA might begin to degrade at processing temperatures exceeding 200 °C, resulting in poor mechanical properties of the blends. In order to avoid this obstacle, some of the research in this topic was carried out by solvent mixing [203,204,205]. The main purpose of PLA/PET blending was to obtain a reaction product of both polymers in the form of a PET copolymer with the possibility of enhanced hydrolytic degradation, which could accelerate or reduce the PET waste disposal problem. The mechanism of the proposed reaction requires the use of dibutyl tin oxide (DBTO) as a catalyst. The reaction of PET/PLA solvent mixtures in different weight ratios was carried out in o-nitrophenol at temperatures ranging from 140 °C to 170 °C and time from 8 to 25 h. PLA was removed from the final product using the chloroform for washing. DSC analysis confirmed the presence of PLA segments in the structure of the resulting block copolymer. Additionally, the PET-*b*-PLA-*b*-PET block copolymer product is characterized by an increased hydrolytic degradation level which was the main goal of the research work. Crystallinity kinetic measurement confirmed the higher crystallinity of this type of copolymer, which may indicate the plasticizing effect of PLA segments, further facilitating the formation of crystallites by increasing the mobility of the chain structure. The length of the aliphatic and aromatic PET-*b*-PLA-*b*-PET segmented block copolymer structures were found to have a great effect on the nucleation mechanism and overall crystallization rate [203]. The nucleation mechanism and rate were significantly affected by incorporating PLA into the copolymer structure, hence affecting the overall crystallization energy barrier.

In the case of solvent mixtures without the use of transesterification catalysts [205], calorimetric studies indicate the partial miscibility of polymers, which confirms the existence of a single glass transition temperature. However, the analysis of the crystallization kinetics showed large differences in crystalline phase growth in favor of PET. The higher crystallization temperature initiates this phenomenon of limited PLA crystallinity, while the main reason for that is the greater tendency of PET to form rigid amorphous fraction (RAF) areas, like crystalline phases, effectively blocking the growth of spherulites of the second polymer. In practice, the solvent method can be used for electrospinning the non-woven fibers. Different PLA/PET blend formulations were used during a spinning procedure [206], and the phase separation was confirmed by the appearance of two separate T_g_ peaks. The addition of PLA reduces the amount of solvent after the spinning process. The shrinkage of the cold crystallized fibers is limited, and the formation of the crystallites measured by WAXS confirmed the oriented structure even in relaxed fibers. The analysis of the results leads to general conclusion of that PLA/PET blends could be used to produce non-woven fibers. 

**Figure 13:**
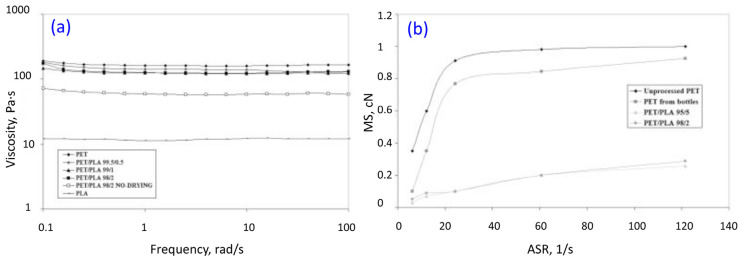
The viscosity and melt strength (MS) comparison between pure PET and its blends with small amounts of PLA. Reproduced with permission from Ref. [210]. Copyright 2012 Elsevier.

For PLA/PET blends obtained by the melt blending technique, the main purpose of the research work is to assess the miscibility and possible degradation of the prepared mixture [207,208,209]. Another possible aspect that is often taken into account in the case of PLA/PET blends is their unintended presence in PET recycling products [210,211]. Due to similar uses and appearance, the collection of separate PET and PLA wastes is difficult, and the presence of PLA in PET recyclables may be a rule in the future. In selected areas of PET processing, this may be a serious problem that limits the potential for PET products to be recycled, especially in high demanding processes such as the production of bottles by injection/stretch blow molding. The addition of PLA in a small amount from 0.5 to 5% does not cause significant changes in the mechanical properties of the resulting blend [210]. These changes are mainly caused by a slight increase in the proportion of the crystalline phase. However, the analysis of the rheological characteristics indicates a visible change in viscosity even for a PLA content of 2% (Figure 13). In practice, this change is even more significant and can be seen after analysis of the melt strength and breaking ratio. The addition of even a low amount of PLA could lead to difficulties during the film, fiber extrusion, and bottle molding. The unfavorable impact of PLA addition necessitates the development of methods for the detection of contaminations in PET processing. One possible variant is the use of in-line methods such as optical spectroscopy. The proven effectiveness of this method [211] indicates the possibility of installing this type of system in the injection nozzle. As observed from the analysis of NIR spectra (near infrared reflectance), not only PLA inclusions can be detected, but also the exact quantity, even below 0.1%. 

From all of the engineering polyesters, PET is the most widely used material, but this popularity is mainly due to its use in the packaging industry. When it comes to the engineering applications, poly(butylene terephthalate) (PBT) is the most commonly used polyester resin. This difference results primarily from the different behavior of these two polymers at the cooling stage of processing. The slow crystallization of the PET resin is very useful during the production of transparent packaging; however, this feature is unfavorable during the injection molding of enginering plastics. That is the main reason for using PBT, as its crystallization speed is higher and makes it easier to achieve highly crystalline structures, despite the fact that the mechnical properties of crystallized PET parts are higher. As in the case of combining PLA with PET, PLA/PBT blends also do not exhibit miscibility, but many research examples have confirmed the self-compatibilization effect. The examples of that phenomena are presented in the works of Di Lorenzo et al. [212,213], where unmodified PLA/PBT blends showed a tendency to interact with the functional groups of both polymers. The effect of this interaction is the formulation of a favorable co-continous morphology which positively influenced the mechanical properties of the blend. Interestingly, even at low concentrations of PBT resin, the influnce of the dispersed fractions did not affect significantly PLA properties. However, already at a PBT content of 40%, the phase morphology system was significantly changed. PLA is transferred into a dispersed phase, but also contains finely dispersed PBT within the droplet. These changes significantly influence the thermomechanical properties and deformation characteristics, from brittle fracture for PLA samples to ductile in PBT-rich blends. The highest improvement was recorded for the elongation value, where the initial value of 3% increased to 160%, while the decrease in tensile strength was visible and dropped from 51 to 33 MPa. The tensile modulus decrease was negligible. In addition to changes in morphology, the PLA/PBT blends exhibited significantly different crystallization kinetics. The composition of the blends did not affect the behavior of PBT, but strongly influenced the PLA crystalline phase formation, where the nucleation of PLA spherulites was accelerated by the presence of already formed PBT spherulites. This mechanism may be an effective method for improving the properties of PLA/PBT blends. Both by accelerating the growth of the PLA crystaline phase, which is a promising processing modification, as well as changing the content of this phase, which translates into an improvement in the mechanical properties. A similar interaction has been observed in studies presented by Samthong et al. [214,215]. However, in this reasearch the same PLA phase nucleation mechanism is subjected to further modification by melt-stretching to obtain fibrillary structured domains of PBT. As a consequence, the kinetics of the PLA crystalline phase forming has been significantly accelerated, which is the result of flow-induced crystallization. In practice, the use of additional fibrillation treatment may strongly affect the nucleation efficiency, reducing the PBT content and shortening the time necessary for the cooling stage. Unlike the other types of PLA-based blends, the self-compatibilized PLA/PBT mixtures do not require additional treatment. However, as reported by other researchers, popular methods of phase compatibilization can be used, such as reactive extrusion with the use of chain extenders [216], or the synthesis of block PLA-PBT copolymers [217]. A significant increase in notched impact strength in PLA/PBT blends was observed with the addition of ethylene–glycidyl methacrylate copolymer-based compatibilizers [218,219,220]. A reduction in the dispersed particle size was observed with the use of the compatibilizer, which proves the decrease in the interfacial tension, suppressing the dynamic coalescence process. 

### 3.2. Polyamides (Nylons)

Due to its excellent mechanical properties, high thermal resistance, and low flammability, polyamides (nylons) became one of the most popular engineering plastics. The availability of many different types of polyamide resins contributes to their extensive use both as injection-molded and extruded materials. Polyamide (PA) is widely used in the automotive industry, where its glass fiber-reinforced composites have successfully replaced elements made up of metal alloys such as the intake manifold, valve cover, or oil pan. Due to the conditions in the engine compartment, only a few polymeric materials meet the technical requirements necessary for these applications, including high and low temperatures, vibration, and contact with numerous chemicals. Biobased polyamide synthesis is currently a very rapidly growing industry, as many polyamide types can be partially or fully based on renewable sources [221]. The first biobased and commercially available polyamide was PA11 synthetized from castor oil. Nowadays, this biobased group also includes PA 10, PA 610, and many other partly biobased polyamides. The ability to synthetize polyamides from vegetable oils and the very good mechanical properties of polyamides are the reason for the growing interest in mixing them with other biopolymers. In the case of mixtures based on PLA, the addition of polyamide allows to improve many of the disadvantages of PLA. Another important feature is that due to the possible biobased origin of both polymers, the use of such mixtures contributes to a decrease in the demand for petroleum-based products. 

**Figure 14:**
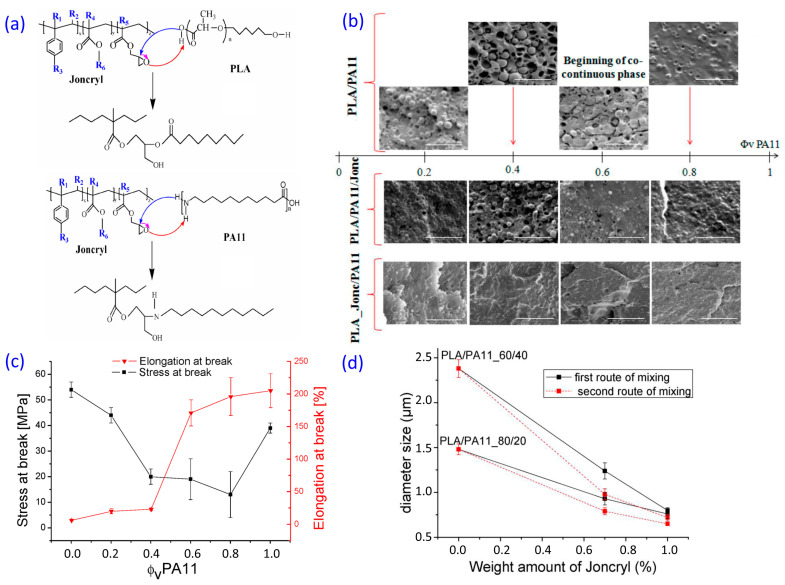
(**a**) Predicted possible reaction between carboxylic end group of PLA and Joncryl chain extender and between amine end group of PA11 and Joncryl chain extender. (**b**) Evolution of the morphological structure of PLA/PA11 blends with and without Joncryl chain extender as a function of the composition. (**c**) Stress at break and strain at break of PLA/PA11 blends at various concentrations. (**d**) The average diameter of the dispersed particles versus the concentration of Joncryl chain extender. Reproduced from ref. [229]. Copyright 2015 MDPI.

In the current scientific studies covering the subject of PLA/PA blends, the main direction of the research is focused on improving the key mechanical properties. The first studies in this topic indicate the lack of important features, like impact strength, elongation at break, or heat resistance, which exclude the use of described blends [222,223]. Because the main reason for these poor properties is the immiscibility of PLA/PA blends, the improvement in their interface interactions has been the subject of many research studies, similar to other polyamide-based blends [224,225,226]. The compatibilization strategies mostly include an increase in the physical interaction or considering the chemical bonding. Due to the low polarity of the PLA chain structure, the use of a reactive approach seems to be more effective, which has been confirmed in the many studies. The use of reactive chain extenders has proven to be especially effective in this area of research. The first attempts to use this type of modifiers were caused by the thermal degradation of the PLA resin during melt blending with PA6 at high temperatures [227]. The use of chain extenders allows for proper mixing operations, which would also cause processing problems during standard extrusion blending. The use of reactive blending indicates the different efficiency of the used chain extenders. Among the already investigated reactive modifiers, multifunctional epoxy-based chain extenders are the most effective, compared to the other types of reactive agents [227] or even pure epoxy resin [228]. In the case of reactive extrusion of PA-based materials, the high melting temperatures are more beneficial, since the effectiveness of the chemical reaction increases. Also, the necessary amount of the modifier usually does not exceed 1%, which lowers the final cost of the compatibility process. In selected cases, the use of chain extender translates to an improvement in the mechanical properties, which is mostly caused by the increase in the molecular weight of the PLA. This improvement is noticeable especially for the ‘elongation at break’ factor, and a similar increase applies to impact strength. With respect to the properties obtained for pure PA6, PLA-based blends are still far below the desirable values. However, in the research works so far, the main goal for the preparation of PLA/PA blends has been increasing the share of the biobased content, which has led to a 70% share of PLA in the mixture. Thanks to the possible synthesis of biobased PA, the research works can be more focused on the optimization of the mechanical properties. This can be made possible by a more balanced blend composition and the use of rubbery impact modifiers, which are very popular for the modification of PA-based engineering blends. In some selected cases, the impact resistance can be improved without the addition of any rubbery particles because some of the properties of biobased polyamides are similar to the behavior of elastomers, which makes it possible for PAs themselves to behave like impact modifiers in PLA. One example of such a modification system is the use of PLA/PA11 blends [229]. For this polyamide type, due to the relatively low melting point, the use of the chain extender is mainly caused by the need to increase the phase interactions. The reactive epoxy functional group from the chain extender or GMA groups react mainly with the COOH end groups of PLA, and the NH_2_ and COOH end groups of PA11 (Figure 14a). The results of the structural observations and measurements of the mechanical properties confirmed the high reactivity of the epoxy-based chain extender (Figure 14b). In the case of the fracture mechanism, this is a decisive criterion for increasing the tough-ness of the prepared blend, as can be seen in the increase in the percentage elongation at break of the blends (Figure 14c). This increasing phase adhesion translates into a better dispersion of the fine rubbery phase and a reduction in the droplet size (Figure 14d). Similar trend changes are observed for PLA blends prepared with the addition of PA6,10 where the modifying agent is liquid epoxy resin [228]. The efficiency of this modification method indicates a lower reaction intensity; however, like in the other examples, it confirms the crucial influence of the interface strength on the morphology of the prepared binary systems. 

**Figure 15:**
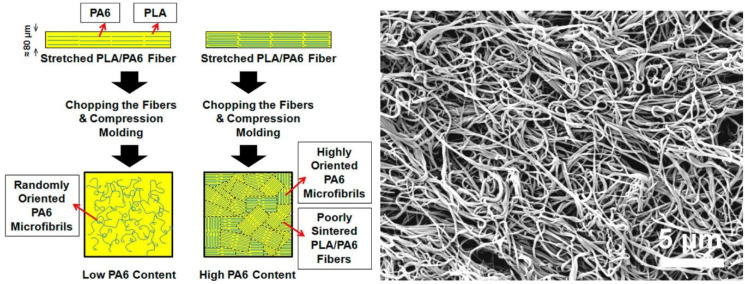
(**Left**) Schematic demonstration of the differences between the microstructures of compression-molded in situ microfibrillar composites with low and high contents of PA6 microfibrils. The poor level of sintering between the chopped PLA/PA6 fibers with a high content of PA6 microfibrils is emphasized using dashed lines. (**Right**) SEM micrograph of a fractured surface of PLA/PA6:97/3 wt% in situ microfibrillar composites after the surface etching of PLA matrix. Adapted with permission from Ref. [235]. Copyright 2015 American Chemical Society.

The compatibilization of the blend interphase using the chemical reaction approach is an efficient and relatively easy to perform modification. However, as confirmed by many studies, it is necessary to use a large share content of the second component to balance the negative impact of the PLA phase on the performance of prepared blend. This condition also applies to polyamide-based mixtures. For this reason, the current research direction attaches much greater importance to the possibility of obtaining a favorable morphology of the prepared materials. The favorable morphology could translate into the ability to improve the overall thermomechanical properties, but also could lead to a possible increase in the PLA share in the blend. An example of deliberate structure modification is the attempt to obtain a microfibril morphology [230,231,232,233]. This concept is based on the melt spinning of the resulting polymer mixture to form the oriented fibril of the dispersed phase. This processing treatment is usually performed during the cold stretching of the partially cooled extruded strand. The prepared strand can be pelletized and formed to the final product by injection or the compression molding technique. The morphology of the prepared pellet is equivalent to co-extruded long fiber composites, where glass fibers are replaced by microfibrils of one of the blend ingredients. It is crucial in this approach to optimize the parameters of the processing of the final article, because the favorable orientation of the microfibril structure can easily be destroyed by excessive heat and shearing during the molding stage. Previous studies dealing with this subject were mainly focused on obtaining the single-polymer composite structure, mostly from popular polyolefins or polyesters [232,234]. However, the principle of selecting a similar type of polymer is not a requirement; an example of that is the research work presented by Kakroodi et al. [235], where the microfibrillar structure of PA6 was used to modify the PLA matrix (Figure 15). In this case, even the small addition of PA6 fibrils below 10% resulted in a significant improvement in the mechanical characteristics, while a further increase of up to 25% did not significantly change the effects obtained at low concentrations of PA6. An explanation of this non-typical behavior is the predominance of structural changes within the PLA matrix over the enhancement effect achieved by increasing the PA6 content. A low content of oriented microfibrils makes it easier to achieve a homogenous structure. Additionally, the small size of of the fibrillar PA6 domains with a diameter of about 200 nm and their very uniform dispersion resulted in a significant increase in the crystallization kinetics, even for samples containing 3% PA6 in the structure. The most visible improvement was related to the elongation at break value, while strength and stiffness were also visibly higher. It is expected that a further increase in the PA6 content would result in a dramatic decrease in the mechanical performance, mainly due to the lack of consistency of the compression-molded samples. An excessive number of fibrils in the blend significantly increases the viscosity, which in consequence prevents effective bonding and obtaining the compacted structure. The formation of a strong fibrillary structure at a low dispersed phase content is a very beneficial feature of the PLA/PA6 blend. In practice, the low content of the fibrils allows the use of the injection molding process, which is much more efficient than compression molding. 

In situ fibril formation (micro or nanofibrils) in a minor component of PLA/polyhydroxyalkanoate (PHA) blends was achieved through simultaneous orientation and shear-induced crystallization of polymer fibers during extrusion in a single step [236]. Shear-induced crystallization facilitated the immediate formation of PHA nanofibers upon applying a high shear rate and elongation strain, eliminating the need for subsequent cooling to initiate crystallization. The PHA fibers, with a diameter of approximately 200 nm, served as both reinforcement and toughening elements, which provided continuous nano-bridging ahead of the crack front, enhancing both the strength and plasticity of PLA. 

**Figure 16:**
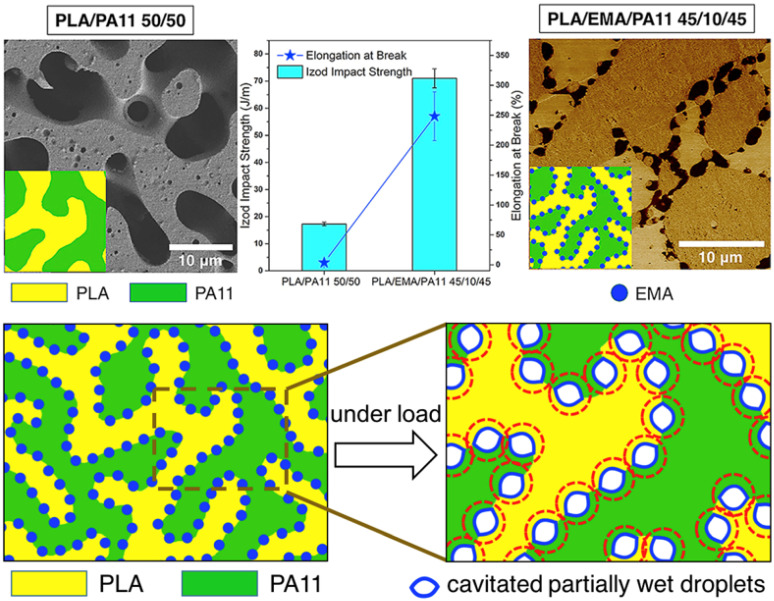
Schematic of the toughening process in PLA/EMA/PA11 with cavitated partially wet droplets percolating the PLA/PA11 interface throughout the entire blend. Adapted with permission from Ref. [238]. Copyright 2017 Elsevier.

**Figure 17:**
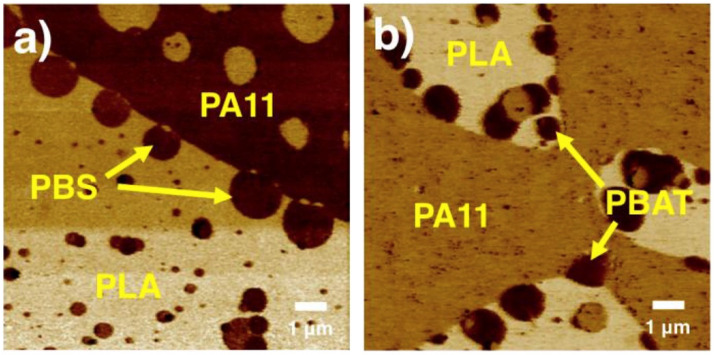

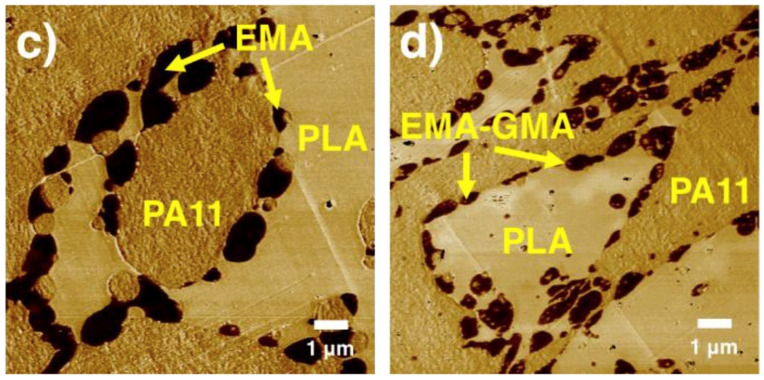
AFM micrographs of (**a**) PLA45%/PBS10%/PA45%; (**b**) PLA45%/PBAT10%/PA45%; (**c**) PLA45%/EMA10%/PA45%; (**d**) PLA45%/EMA-GMA10%/PA45%. Adapted with permission from Ref. [238]. Copyright 2017 Elsevier.

**Figure 18:**
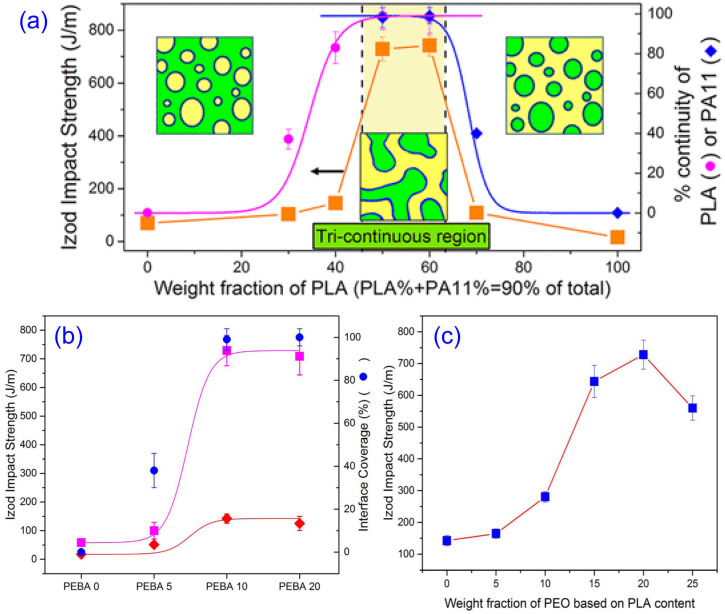
(**a**) Significant increase in impact strength when all three phases are fully percolated with the addition of polyether-b-amide (PEBA) into a PLA/PA11 blend. (**b**) Impact strength as a function of PEBA content in PLA/PEBA/PA11 where the PLA:PA11 ratio is maintained at 1:1 (◆) and PLA(PEO)/PEBA/PA11 with 20% PEO (based on the PLA content) and a PLA:PA11 ratio of 1:1 (■). Also shown is the PEBA interfacial coverage (●) in both the ternary and quaternary systems as a function of PEBA content. (**c**) Impact strength as a function of PEO content in PLA(PEO)PEBA/PA11 with 45PLA(xPEO)/10PEBA/45PA11. Reproduced with permission from Ref. [239]. Copyright 2017 American Chemical Society.

The favorable structural changes may also lead to a significant increase in the impact resistance of the resulting PLA-based mixtures, which is difficult to achieve using the simple blending approach. The most popular and effective modification method for increasing the impact strength is the addition of an elastomer phase. However, in the case of ternary blends, the performance of this method may be reduced due to the unfavorable location of the rubbery phase. Deposition in only one of the matrix components can lead to a decrease in the blend toughness. For this reason, for ternary blends the best results for improving the toughness are obtained for systems where the elastomer phase fully or partially wets the surface of the selected matrix component. This principle works for the blend systems containing polyamides and polyolefins [237]. An evaluation of this toughening system for biobased PLA/PA11 blends is described by Zolali et al. [238,239]. The presented research work confirms the possibility of achieving a co-continuous structure of a 50/50% blend of these polymers, and the impact properties are highly improved after introducing the elastomer phase. It is important to locate the dispersed rubbery phase at the PLA/PA11 interface [238]. During the application of dynamic loads, the deformation mechanism allows for increased shear yielding due to the intense cavitation of the elastomer particles at the PLA/PA11 blend interfaces, as depicted in Figure 16. As confirmed by the study, this phenomenon is observed only for selected blends containing ethylene methyl acrylate copolymer (EMA) and ethylene methyl acrylate-glycidyl methacrylate (EMA-GMA) modifiers. When poly(butylene succinate) (PBS) and poly(butylene adipate-co-terephthalate) (PBAT) are used as the impact modifiers the impact improvement is less visible, which can be due to the very low interfacial tensions between PLA/PBS and PLA/PBAT in the entire blend [238]. For PBS and PBAT, a large share of the droplets is distributed evenly inside the PLA phase (Figure 17a,b). For the EMA and EMA-GMA modifiers, the concentration of the droplet at the interface exceeds 90%, leading to almost complete wetting of the PLA/PA11 interface, and displaying more symmetrical droplets at the interface due to their similar affinity towards both PLA and PA11, as demonstrated in Figure 17c,d. The beneficial effect of the interface wetting system is confirmed by the increasing impact strength of the blend, while maintaining the same strength and slightly lower stiffness. In the case of the described morphology of the structure, it is possible to further increase the impact strength by completely wetting the PA11/PLA interphase, which is presented by Zozoli et al. [239], where a PLA/PA11 blend was modified by the addition of the poly(ether-b-amide) (PEBA) elastomer. The resulting ternary blend PLA/PA11/PEBA (45/45/10%) structure was characterized by a high improvement in the impact strength compared to pure PA11, from 70 J/m to 140 J/m, while maintaining a higher modulus and tensile strength. The obtained blends have undergone further modification by the addition of poly(ethylene oxide) (PEO), modifying the PLA impact, which resulted in an increase in impact strength to over 700 J/m, but the consequence of this was a significant reduction in other mechanical characteristics (Figure 18). Grafted polymers containing reactive chemical groups such as maleic anhydride and isocyanate, along with reactive chemicals like peroxides and metallic-based catalysts, have shown significant potential in enhancing the compatibility of PLA-based blends [173].

### 3.3. Polycarbonate—PC

Unlike the other popular engineering plastics, polycarbonate (PC) is one of the few examples of an amorphous polymer. In addition, this polymer combines many good mechanical properties, not available for other thermoplastics, such as high transparency, dimension stability, and low flammability. Despite its high price, all these features make PC very difficult to replace with other engineering polymers, which is the main reason for the popularity of PC-based blends. For most of the polymer blends, PC is still the main popular component of the mixture, as it is used to maintain good impact strength and dimension stability. The most common used type of the PC-based blends is the PC/ABS mixture. Despite its worse overall thermomechanical properties, the addition of acrylonitrile-butadiene-styrene terpolymer (ABS) leads to a reduction in the price and an increase in the processability. Rarely mentioned advantages of PC/ABS blends include the ability to print and galvanize weld with other polymers. PC is also blended with engineering polyesters like PET [240] or PBT [241]. The addition of PBT also provides higher chemical resistance and thermal stability. Similar advantages are provided by the addition of PET, with additionally improved surface properties such as high gloss and low coefficient of friction. The addition of both polyesters greatly reduces the viscosity of PC. The main disadvantage of all mixtures compared to pure PC is the compromised transparency.

**Figure 19:**
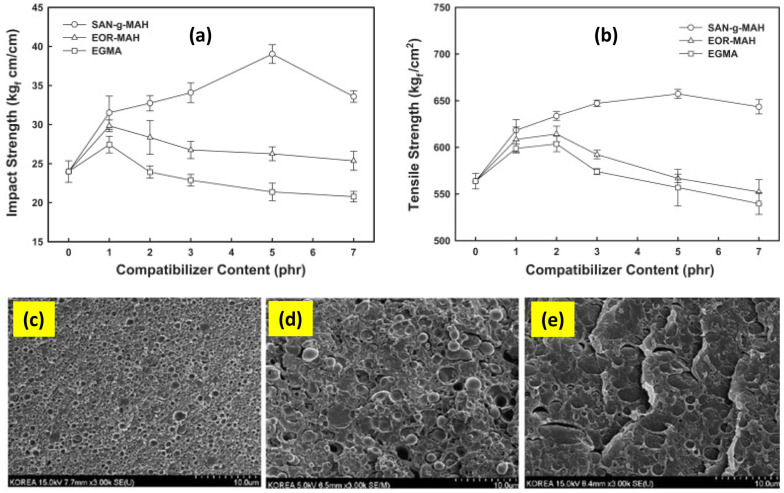
Top row: the mechanical properties of PC/PLA blends (70/30) after addition of different types of compatibilizers. (**a**) Impact strength and (**b**) tensile strength. Bottom row: scanning electron micrographs of the PC/PLA (70/30) blends with the 5 phr of compatibilizer: (**c**) SAN-g-MAH; (**d**) EOR-MAH; (**e**) EGMA. Reproduced with permission from ref. [247]. Copyright 2011 Elsevier.

The current attempts to increase the biobased content of PC-based blends are mainly focused on the addition of different types of biobased polyesters. The best example for that is the replacement of PBT by partially biobased poly(trimethyl terephthalate) (PTT) [242,243,244,245,246]. PTT resin contains up to 30% of biomass-based renewable material, and most of the basic properties are comparable to PBT, which also applies to PC/PTT blends. Despite the lack of miscibility of both polymers, it is effective to use the reactive blending technique to successfully increase the phase interactions and improve the properties of PC/PTT blends. The further reduction in fossil fuel use would be possible through the replacement of PTT by fully biobased PLA resin. Unfortunately, the relatively good properties for PC/PTT blends do not apply to PLA, despite the use of similar modification methods. Numerous researchers dealing with this issue have focused on phase interactions between PC and PLA domains, which was most often attempted by the addition of different types of reactive compatibilizers. An example for that approach is the research presented by Lee et al. [247], where the authors are comparing the effects of several compatibilizers on the properties of PC/PLA mixtures. The investigated PC-rich blends, with a PLA content of 30%, was melt-blended with three types of modifiers. Poly(styrene-g-acrylonitrile)-maleic anhydride (SAN-g-MAH), poly(ethylene-co-octene) rubber-maleic anhydride (EOR-MAH), and poly(ethylene-co-glycidyl methacrylate) (EGMA) were added with a variable share from 1 to 7%. The mechanical properties of the prepared blends confirmed the high efficiency of SAN-g-MAH (Figure 19a,b). The application of other modifiers did not change the properties compared to the unmodified PC/PLA samples. The structural observations confirmed the significant improvement in the SAN-g-MAH containing blends, where the diameter and dispersion level of the PLA phase have undergone a favorable change over the other modified structures (Figure 19c–e). The interfacial tension measurements also indicate the effectiveness of interphase modification, which clearly confirms the successful compatibilization of PC/PLA blends. 

A slightly different approach to PC/PLA blend modification is presented by Hashima et al. [54]. Instead of focusing on the compatibility of the major PC and PLA phases, the main objective of the study was to improve PLA properties by introducing hydrogenated styrene-butadiene-styrene block copolymer (SEBS) as the rubber impact modifier. Another component, poly(ethylene-co-glycidyl methacrylate) (EGMA), was used to increase the dispersion of the elastomer phase by reactive compatibilization. Structural observations confirmed the reduction of SEBS rubber droplets, which has resulted in significant improvement in the impact resistance. The addition of polycarbonate to the blend leads to the reduction of a necessary amount of the elastomer phase and an increase in the thermal stability. The attempts to increase PLA impact resistance usually require the use of an effective compatibilization of PLA and the used elastomer phase. So far, the most effective method of improving the adhesion between immiscible phases was the use of reactive melt blending in presence of chain extenders (CE). The effectiveness of this method in the case of polyester-based materials is confirmed by the successful use of highly reactive epoxy-based CE [248]. In the case of PC/PLA blends, this method is also used, which is additionally caused by the necessary limitation of PLA chain thermal degradation. The decomposition of the temperature-sensitive PLA resin is inevitable during melt-mixing with PC, which has processing temperatures close to 300 °C, higher than other engineering polymers like PA6, PBT, or PET, making processing more problematic. The use of the popular epoxy functional styrene acrylic copolymer CE (Joncryl 4368 type from BASF, Ludwigshafen, Germany) was presented by Srithep et al. [249] For PC/PLA blends containing 30 and 50% of PLA resin, the processing temperature during the reactive extrusion step reached 240 °C. The results of the gel permeation chromatography (GPC) confirmed the possibility of CE reaction between the PC carboxyl and PLA hydroxyl groups, which may lead to the formation of PC-PLA copolymers, which is partially confirmed by the significant change in the molecular weight and polydispersity index of the CE-modified blends. In practice, the improvement in the mechanical properties of modified PC/PLA blends is mainly related to the elongation improvement. Moreover, the addition of CE was also found to be able to increase the thermal stability of the blends (measured by heat deflection temperature (HDT)). The results of the DSC measurements clearly confirmed the decrease in the phase crystallinity of the PLA, which suggests that the main reason for the improvement is a significantly higher molecular weight and a decrease in the mobility of the PLA chains. As a result of that, the addition of around 1% CE increased the HDT temperature of PC/PLA (50/50%) blends from 60 °C to over 100 °C. 

A similar strategy of using reactive blending in the presence of CE was used to prepare impact-modified PC/PLA blends [55], where the toughness was improved with the addition of an acrylic rubber core-shell modifier. The high efficiency of this type of modifier was reported in many PLA applications and confirmed for reference samples in studies already discussed. As discussed previously, the annealing process increases the PLA crystalline phase content, leading to an even higher enhancement of the impact strength. This favorable change for PLA does not translate into a visible increase in the toughness of PC/PLA blends. The expected difference is caused by the small PC/PLA interface interaction. Additional chemical treatment by subjecting the blend to reactive blending using CE leads to significant changes in morphology and properties. A visible change can be observed even for PLA-rich samples containing only 10% of PC and additionally 5 phr of the rubbery modifier, while increasing the PC content leads to a further increase of the impact resistance. A similar improvement could be observed for HDT temperature changes, where increasing the PC content and the annealing treatment has the largest influence in the favorable trend, since the modification of pure PLA by addition of the elastomer phase leads to a decrease in the heat resistance, even after annealing where the crystallinity level is increased. The beneficial effect of CE increasing the interactions between PC/PLA is further enhanced with heat treatment, which increases the PLA crystallinity. The additional modification of PLA using the impact modifier allows us to obtain high impact toughness and heat-resistant blends. 

Because the proportion of the crystalline phase significantly impacts the overall properties of PLA itself, in the case of PC/PLA blends, the high crystallinity of the PLA phase should be considered as a key factor for improving the overall properties of the blends. For mixtures containing PC, the high thermal resistance and favorable mechanical properties can be significantly reduced by the addition of low crystalline PLA. This subject is discussed in the research works presented by Lin et al. [55,56]. 

**Figure 20:**
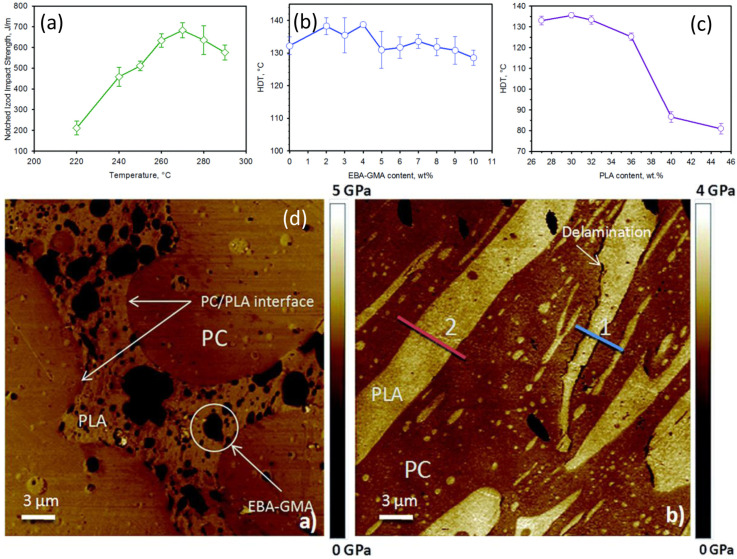
(**a**) Overall toughness of PC/PLA blends containing 32 wt% of PLA and 6% EBA-GMA, blended at different temperatures. (**b**) The effect of impact modifier loading on heat deflection temperature of PC/32 wt% PLA blends. All samples were prepared at 270 °C. (**c**) The effect of PLA content on the heat deflection temperatureof PC/PLA/EBA-GMA blends. The amount of EBA-GMA was 20% of the amount of PLA in all blends. All samples were prepared at 270 °C with 0.3 phr of chain extender. (**d**) Atomic force microscopy images (AFM) of (**d**(**a**)) impact-modified 32 wt% PLA/62 wt% PC/6 wt% EBA-GMA blend (**d**(**b**)) non-modified 32 wt% PLA/68 wt% PC blend, the colored lines are indicating the positioning of the E modulus profile scanning. All samples were prepared at 270 °C. Adapted with permission from Ref. [251]. Copyright 2016 RSC.

Previous studies on the use of reactive chain extenders have confirmed the successful compatibilization of multicomponent blends. In the particular case of PC/PLA mixtures, the desirable effect in terms of impact strength improvement is achieved by the additional dispersion of the elastomer impact modifiers. However, the evaluation of the HDT indicates a decrease in heat resistance, which is the result of increased molecular weight leading to limited PLA crystallinity. The visible improvement in heat resistance is achieved by the thermal treatment during the annealing process, but still, the observed change is usually less pronounced than for unmodified samples. The most used chain extenders are multifunctional copolymers containing many reactive epoxy functional groups. In most of the research studies dealing with the subject of the reactive blending process, the preparation methodology did not consider the significant influence of the processing conditions. Few works discuss the effect of time or the mixing procedure [250], but usually the evaluation of the melt conditions are not considered as the main subject of the research. This important topic in the context of PC/PLA blend preparation was discussed by Yuryev et al. [251], where the main research interest was focused on the thermal conditions during the melt processing. The possibility of high temperature reactive extrusion was studied, not practiced before due to the low thermal resistance of PLA. The results of the studies conducted at high temperatures indicate a much higher efficiency of the reaction kinetics. This favorable processing treatment is attributed to the increased reactivity of the used multifunctional epoxy-based chain extender (Joncryl ADR-4368), whose partial decomposition above 250°C and more intensive scission of the polymer chains results in improved reaction density at the interphase. The composition of the prepared blend was dominated by PC, while the PLA content was mostly limited and did not exceed 40%. The composition was supplemented by the rubber impact modifier poly(ethylene n butylene acrylate glycidyl methacrylate) copolymer (EBA-GMA). The use of the PC-rich blend system allows us to limit the amount of impact modifier required for PLA modification. As shown in Figure 20a from [251], the notched impact strength of PC/PLA blends with 32 wt% of PLA and 6% of EVA-GMA increased with temperature until it reached a peak, then decreased. The highest toughness was achieved using CE at 270°C. This temperature is much higher than the conventional processing range for PLA (180–220°C), and there is limited knowledge about PLA’s behavior at such elevated temperatures. The samples were not subjected to the annealing procedure; however, they still exhibited very high HDTs, similar to pure PC (Figure 20b). There is also no visible heat resistance deterioration caused by increasing the rubber content. The apparent decline occurs only after reaching a PLA content of 40% (Figure 20c). The reason for this phenomenon is the location of the rubber phase droplets within the PLA domains, which limits its potential negative influence on the thermal properties of the PC phase. The favorable morphology of the three-phase structure was confirmed by AFM mapping, where there was visible accumulation of the EBA-GMA phase inside the PLA phase, which indirectly confirms the improved efficiency of the high-temperature reactive compatibilization (Figure 20d).

The durability of polymeric materials is a particularly important issue for engineering materials, where the stability of properties over a long period of time determines the possible applications, especially for such demanding industries like the automotive industry. For polymer blends containing PLA, it is even more important to evaluate the change in basic properties in function of time, since the main feature of PLA is the tendency to decompose. The degradation of PLA is therefore a major contradiction when most of the engineering applications require the maintenance of stable mechanical properties. In the case of PC/PLA blends, the stability of the material will depend mainly on the PC content, while the resistance of the PC resin to elevated temperatures and the presence of water is significantly higher than that of PLA. However, this does not mean that the specific conditions are not affecting the properties of PC-based blends, since at the high melt processing temperature the PC chains are highly sensitive to hydrolytic degradation. However, this problem does not occur at normal operating temperatures due to low water absorption. The durability of PC/PLA blends was the topic of the research presented by Harris et al. [132], where blends with various PLA contents have been subjected to accelerated climate tests. The most popular durability tests used in the automotive industry reflect the conditions of the car cabin interior, where exposure to sunlight leads to intensive warming and can be connected with possible elevated humidity, causing highly demanding operating conditions. Most typical tests are carried out at a temperature of 70°C and a high relative humidity, close to 100%. In the discussed study, the aging was conducted over a period of 28 days, which is the equivalent of about 4 years of aging in real conditions. Parts of the samples were subjected to control measurements after every 7 days. All of the tests confirmed the negative influence of the aging process, especially for PLA-rich samples, which is the expected result. The more interesting changes were related to the intensive hydrolytic degradation of the PC phase, which did not occur for the reference PC/ABS samples. The main reason of the accelerated PC decomposition is the presence of PLA decomposition byproducts, mainly carboxylic acid. The presence of a low pH environment increased the speed of hydrolytic degradation of the PC phase. This intensity of PC degradation is high enough to change the nature of the macromolecular morphology from amorphous to semi-crystalline, which is detected from the DSC analysis. 

### 3.4. Other Polymers

The enhancement of PLA’s properties by blending with engineering polymers is a fairly widespread topic in the literature. The presente review is manly focused on PLA blends with the most commonly used engineering polymers, which does not change the fact that this subject covers a much larger group of plastics. This paragraph provides some examples of the use of PLA in mixtures with less popular engineering polymers. In the case of polymers described herein, such as POM, PMMA, or PTFE, the lower popularity does not mean worse properties. The lower consumption of these polymers is primarily related to higher price, processing difficulties, or limited availability on the market. However, this does not change the fact that these polymers often have very specific properties needed in many applications. The described research works have intended to use these selected properties, or at least, not lose them with the increasing PLA content. 

#### 3.4.1. Acrylonitrile Butadiene Styrene

Among the commercially available amorphous plastics, acrylonitrile butadiene styrene (ABS) is one of the most important polymers used in engineering applications. It is worth noting that the main factor in this case is not the transparency, as in the case of PMMA, PS, or SAN resins, but a very favorable balance of strength, toughness, and heat resistance of ABS-based materials. Because of its two-phase structure, most ABS grades are opaque. This feature is a consequence of phase separation, where the SAN copolymer is the matrix for finely dispersed polybutadiene rubber. This structure is characterized by excellent impact properties, which is the basic difference from other brittle styrene-based resins. Due to the combination of good mechanical properties, a relatively high operational temperature range, and dimensional stability, ABS is one of the most popular materials used in the manufacture of electronic equipment or car body parts. Apart of numerous advantages, this polymer is also often used as a component of polymer blends. In this category, the most popular species are PC/ABS blends. The perspective of using ABS as a component for PLA-based blends is therefore a natural consequence of the popularity of the ABS resin. 

Previous research studies have confirmed the lack of miscibility of these two polymers, which leads to the need for an effective compatibilization process. An example of such a modification is presented in the study of Li et al. [252]. An impact modifier in the form of a SAN-GMA copolymer was introduced to the PLA/ABS blend, and in addition, a small amount of the ethyltriphenylphosphonium bromide (ETPB) catalyst was used to enhance the reaction efficiency. The effectiveness of this type of modification was confirmed by very good impact resistance results; the impact strength was doubled after the addition of the SAN-GMA copolymer, while the further addition of the ETPB catalyst increased the toughness even more. Glycydyl methacrylate rubber was also used as a impact modifier in a study by Sun et al. [253]. However, in order to simplify the methodology of mixing, the GMA was grafted onto the polybutadiene to form an ABS-g-GMA copolymer. This facilitated the PLA-ABS interface compatibilization, eliminating the need for additional reaction catalysts. As a consequence of this process, the 1% content of GMA in the ABS-g-GMA copolymer resulted in an impact strength increase from an initial 20 J/m to 540 J/m. A PLA content of 70% indicates the efficiency of this method compared to other reported techniques. The possible reason was the core-shell structure of ABS-GMA structures and the favorable location of the grafted GMA. The issue of PLA-ABS interface compatibilization was the subject of study for other researchers [254,255,256,257], where different types of reactive compounds were sucessfully used. 

#### 3.4.2. Poly(methyl methacrylate)

The main area of application for poly(methyl methacrylate) (PMMA) includes products that require high transparency. In this category, the characteristics of PMMA are the best in relation to other polymers, even those that are fully amorphous. PMMA, also known as acrylic glass, is characterized by excellent transparency. In addition, it does not show reduced resistance to sunlight, and is characterized by quite high thermomechanical resistance: above 100 °C in HDT tests [258,259,260]. For PLA/PMMA blends, immiscibility is observed when processed through solvent casting. However, when melt processed in a twin-screw extruder, the two polymers become miscible in the melt state, as indicated by a unique glass transition temperature between PLA and PMMA [261]. The partial miscibility of the blends was also confirmed by Zhang’s work, which investigated blends of amorphous PLA and crystalline PLLA with PMMA [262]. The presence of PMMA significantly inhibited PLA crystallization, with minimal or no crystallinity observed in blends containing more than 30% PMMA [263]. These results highlight the intriguing potential of these blends for various applications, regardless of whether they exhibit phase separation.

Stereocomplex PLA was used as a reinforcement to enhance the physical cross-linking point to significantly restrict poly(methyl methacrylate) (PMMA) molecular chain motion and improve the morphology and overall mechanical properties of sc-PLA/PMMA nanofibers [264]. The addition of stereocomplex PLA to transparent PMMA filters demonstrated a high PM2.5 removal efficiency of up to 99.5%. 

The incorporation of nanosilica can further improve the phase separation temperature of the filled PLA/PMMA blends, suggesting that nanosilica enhances the phase stability of these blends [265]. Additionally, nanosilica increased the entanglement density and decreased the entanglement molar mass of the blends. The molecular entanglement between the miscible amorphous/semicrystalline PMMA/PLA blends is found to positively influence the shape memory performance [266]. The use of nanofillers to enhance various properties of PLA/PMMA blends is appealing. For example, incorporating graphene nanoplates and functionalized graphene can improve electrical conductivity [267].

## 4. Conclusions

In conclusion, the engineering applications of polylactic acid (PLA) demonstrate significant potential for improvement through the strategic preparation of polymer blends. Like its counterparts in the thermoplastic polyester family, PLA exhibits limitations such as low operational temperature and poor impact strength, which are pivotal in engineering contexts. However, leveraging modifications in PLA blends offers a promising avenue for enhancing its mechanical properties.

The interdependence between microstructure morphology and the final properties of the developed PLA and PLA-based composites is significant. This underscores the efficacy of targeted modifications in enhancing both notched impact strength and heat deflection temperatures. Furthermore, the augmentation of PLA phase crystallinity emerges as a crucial aspect in improving its performance, with nucleating agents and impact modifiers playing pivotal roles.

Drawing from established methodologies applied to modify PET or PBT resins, it becomes evident that enhancing PLA blends necessitates increasing the compatibility between major components and addressing the inherent properties of the PLA phase itself. A notable example is the utilization of reactive extrusion alongside functionalized elastomers as impact modifiers, which effectively enhances impact strength. Moreover, when compared to commonly used blends like PC/ABS, PLA blend systems incorporating polyamide or polycarbonate exhibit notably superior properties. 

Despite challenges such as the availability and cost of chemicals and modifiers, the evolving landscape of biotechnology, supported by legislative changes and growing consumer interest in sustainable resource management, holds promise for overcoming these obstacles. As such, the widespread adoption of PLA-based polymer blends in engineering applications will soon appear increasingly feasible.

In essence, the potential for enhancing PLA’s properties through strategic blend formulations, coupled with the momentum in biotechnology and sustainability initiatives, positions PLA as a valuable component in the repertoire of polymer materials for diverse engineering applications. However, blending PLA with petroleum-based engineering polymers decreases its sustainability. Fossil-based polymers are not ideal for enhancing the properties and toughness of PLA. However, this area has seen remarkable improvement. Biobased and biodegradable engineering polymers should be considered as ideal substitutes when blending with PLA to improve their toughness and other engineering properties. Further studies are required in this research direction. 

Summarizing the market potential of the modification methods presented in this review: for PLA-based engineering blends, the coming years will probably still not bring any significant changes in market conditions, which, however, applies to the entire biopolymer industry. The technical plastics market will probably focus on introducing increasingly advanced solutions for recycling petrochemical polymers. The main basis here is the economic aspect and new legal regulations governing the management of polymer products after their service life. However, in the case of the biomedical industry, it can be seen that the growing interest in the concept of regenerative medicine may result in the increasingly widespread use of non-resorbable materials based on PLA copolymers. From an economic point of view, the most promising market for the use of PLA mixtures is the 3D printing industry, where the dominant position of PLA is already noticeable. Interestingly, unlike other polymer processing industries, in the case of 3D printing by extrusion, PLA is the base material with the best available processing characteristics, significantly facilitating the introduction of new modified products to the market. The topic of modification presented in the article, aimed at improving the key material characteristics of PLA-based blends, is very important for expanding the application range of biopolymers.

## Figures and Tables

**Table 1:** Reported annealing conditions and maximum heat deflection temperatures of PLA, along with its blends and composites.

No.	PLA and Their Blends and Composites	Annealing Temperature (°C)	Annealing Duration (Hour)	Reported Max HDT (°C)	Reference
1	PLA	100	3	118	[188]
	PLA/Basalt Powder (95/5)			119	
	PLA/Basalt Powder (90/10)			126	
	PLA/Basalt Powder (80/20)			130	
2	PLA	70, 80, 90, 100	6	~66	[189]
3	PLA	90	5	67	[186]
	PLA/EVA (80/20) PLA/EVA (70/30)			~57	
4	PLA	120	0.5	~56	[190]
	PLA/Bamboo leaf fiber (90/10)			59.3	
	PLA/Coir fiber (90/10)			61.7	
5	PLA	90	3	63	[187]
	PLA/ABS			68	
6	PLA/Methyl methacrylate-butadiene-styrene (MBS) (90/10)	Unannealed	-	53	[191]
		120	0.25	62	
			0.5	72.4	
			1	83.6	
7	PLA	90	5	64	[192]
	PLA/Acrylonitrile-EPDM-styrene (70/30)			63.6	
8	PLA	Unannealed	-	59.7	[158]
		92	0.16	68.3	
			0.33	74.1	
			0.5	78.5	
	PLA/Poly(butylene succinate) (80/20)	Unannealed	-	57	
		92	0.16	75.4	
			0.33	82.6	
			0.5	78.5	
9	PLA	Unannealed	-	56.7	[193]
	PLA/EVM-GMA (80/20)				
		100	0.33	94.2	
